# Integrated omics unveil the secondary metabolic landscape of a basal dinoflagellate

**DOI:** 10.1186/s12915-020-00873-6

**Published:** 2020-10-13

**Authors:** Girish Beedessee, Takaaki Kubota, Asuka Arimoto, Koki Nishitsuji, Ross F. Waller, Kanako Hisata, Shinichi Yamasaki, Noriyuki Satoh, Jun’ichi Kobayashi, Eiichi Shoguchi

**Affiliations:** 1 Marine Genomics Unit, Okinawa Institute of Science and Technology Graduate University, Onna, Okinawa, 904-0495 Japan; 2 Present address: Department of Biochemistry, University of Cambridge, Cambridge, CB2 1QW UK; 3 Showa Pharmaceutical University, 3-3165 Higashi-Tamagawagakuen, Machida, Tokyo, 194-8543 Japan; 4 Marine Biological Laboratory, Graduate School of Integrated Sciences for Life, Hiroshima University, Onomichi, Hiroshima 722-0073 Japan; 5 Department of Biochemistry, University of Cambridge, Cambridge, CB2 1QW UK; 6 DNA Sequencing Section, Okinawa Institute of Science and Technology Graduate University, Onna, Okinawa, 904-0495 Japan; 7 Graduate School of Pharmaceutical Sciences, Hokkaido University, Sapporo, 060-0812 Japan

**Keywords:** Polyketide synthases, Harmful algal blooms, Dinoflagellates, Iso-Seq, Duplication, *Amphidinium*

## Abstract

**Background:**

Some dinoflagellates cause harmful algal blooms, releasing toxic secondary metabolites, to the detriment of marine ecosystems and human health. Our understanding of dinoflagellate toxin biosynthesis has been hampered by their unusually large genomes. To overcome this challenge, for the first time, we sequenced the genome, microRNAs, and mRNA isoforms of a basal dinoflagellate, *Amphidinium gibbosum*, and employed an integrated omics approach to understand its secondary metabolite biosynthesis.

**Results:**

We assembled the ~ 6.4-Gb *A. gibbosum* genome, and by probing decoded dinoflagellate genomes and transcriptomes, we identified the non-ribosomal peptide synthetase adenylation domain as essential for generation of specialized metabolites. Upon starving the cells of phosphate and nitrogen, we observed pronounced shifts in metabolite biosynthesis, suggestive of post-transcriptional regulation by microRNAs. Using Iso-Seq and RNA-seq data, we found that alternative splicing and polycistronic expression generate different transcripts for secondary metabolism.

**Conclusions:**

Our genomic findings suggest intricate integration of various metabolic enzymes that function iteratively to synthesize metabolites, providing mechanistic insights into how dinoflagellates synthesize secondary metabolites, depending upon nutrient availability. This study provides insights into toxin production associated with dinoflagellate blooms. The genome of this basal dinoflagellate provides important clues about dinoflagellate evolution and overcomes the large genome size, which has been a challenge previously.

## Background

Phytoplankton communities are essential components of marine ecosystems, and dinoflagellates are of special interest because they exhibit morphological diversity, high species richness, and the capacity to survive in different ecological niches [1]. They are also infamous contributors to harmful algal blooms (HABs), often producing toxins that are deadly to aquatic organisms and humans [2]. Dinoflagellates exhibit many genetic and cellular features that are highly unusual for eukaryotes. The persistent condensed state of dinoflagellate chromosomes and their liquid crystalline organization, loss of nucleosomal chromatin packaging, use of 5-hydroxymethyluracil in nuclear genomic DNA, and huge genomes of some dinoflagellates (≥ 100 Gbp) are anomalous for eukaryotes [3–5]. Recently, the critical role of tandem-duplicated, unidirectional, single-exon genes to survive in cold, low-light environments was reported in two draft genomes (~ 2.8 Gb and ~ 3.0 Gb) of the free-living dinoflagellate, *Polarella glacialis* [6]. Even with ongoing genomic efforts, understanding of dinoflagellate toxin biosynthesis remains elusive due to their unusually large genomes and limited biosynthetic surveys [4–10].

Toxic compounds associated with HABs have a polyketide backbone, are synthesized by polyketide synthases (PKSs), and can be linked to non-ribosomal peptide synthases (NRPSs), resulting in hybrid molecules [11]. Several evolutionary events have enabled production of novel polyketides and non-ribosomal peptides [12]. To explore molecular mechanisms involved in secondary metabolite biosynthesis, we sequenced the genome of a basal dinoflagellate, *Amphidinium gibbosum*, belonging to a genus associated with HABs [3, 13–16]. *Amphidinium* species (Gymnodiniales: Gymnodiniaceae) possess intricate secondary metabolic pathways that synthesize unique macrolides with unusual, odd-numbered lactone rings, but their biosynthesis has remained unresolved [17–19]. Changes in environmental levels of nitrogen and phosphorus heavily influence the production of toxic metabolites during HABs [20–22], and an understanding of nutrient dynamics is critical to any attempt to understand molecular mechanisms associated with toxin production.

Biosynthesis of secondary metabolites having diverse structures and biological activities depends on environmental stresses and is sometimes restricted to specialized structures. Regulation of toxin biosynthesis tends to be coordinated principally at the transcriptional level [23]. Transcriptome analysis of toxic dinoflagellates has been performed [24], but the regulatory mechanisms involved in secondary metabolism during nutrient stress have not been fully explored. While individual omics datasets offer overviews of static states of dinoflagellate systems, integrating several kinds of datasets can strengthen inferences and preclude false assumptions. By sequencing the *A. gibbosum* genome, transcriptome, and microRNAome, we investigated genomic features and post-transcriptional regulation during nutrient stress, to globally comprehend its secondary metabolism. We identified several miRNAs from the assembled genome and their targets in the transcriptome under phosphate and nitrate starvation. Our integrated omics approach reveals the contributions of repetitive elements and introns in this dinoflagellate genome. It also illustrates the effects of alternative splicing and polycistronic expression and suggests possible implications of miRNA-mediated post-transcriptional regulation of secondary metabolism.

## Results and discussion

### What accounts for the large genome size and genomic features of the basal dinoflagellate, *A. gibbosum*?

**Fig. 1:**
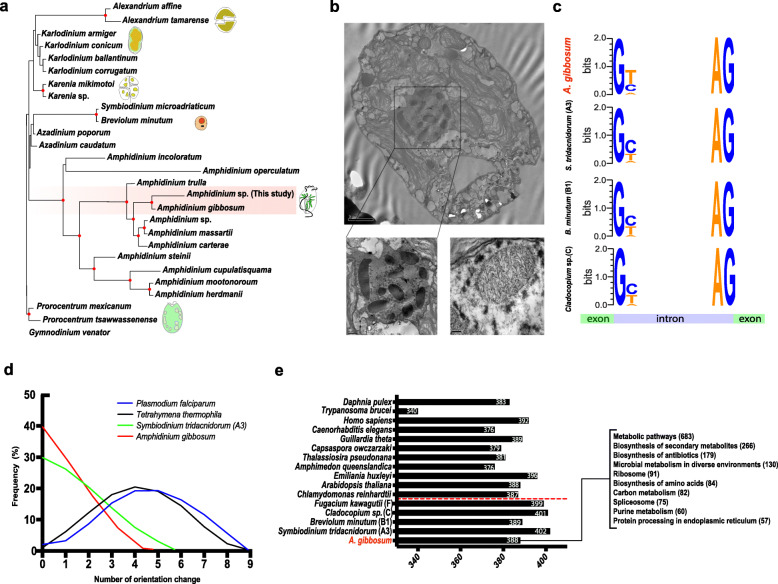
Genomic features of the basal dinoflagellate, *Amphidinium gibbosum*. **a** Phylogenetic analysis of dinoflagellates using partial LSU rDNA sequences by maximum likelihood, with red dots at nodes indicating bootstrap support ≥ 80%. **b** Transmission electron microscopy of *A. gibbosum* with a lower insert showing a detailed region of condensed chromosomes (lower left: ~ 11 chromosomes in nuclei; lower right: a chromosome). **c** Non-canonical splice sites show the use of GC and GA, in addition to GT, at the 5′ donor splice site in *A. gibbosum*, a unique feature of dinoflagellates. **d** Gene orientation changes using a 9-gene sliding window and 9-gene steps confirm the unidirectional alignment of genes in dinoflagellates. **e** KEGG pathways recovered from *A. gibbosum* in comparison with other eukaryotes show biosynthesis of secondary metabolites among top 10 hits. Numbers in brackets indicate the number of enzymes recovered from each pathway category

We estimated that the 6.4-Gb *A. gibbosum* genome (~ 6.4 Gb by flow cytometry and ~ 6.3 Gb by *k*-mer analysis) encodes 85,139 genes, of which ~ 48% had matches in available databases (Fig. 1a, b; Table 1; and Additional file 1: Supplementary Fig. 1a-e, Additional file 2: Supplementary Table 1). The size difference between the estimated and assembled genomes may be due to the liquid crystalline structures of dinoflagellate chromosomes [3–5]. Genomic data showed the utilization of GC and GA (5′ donor splice sites) in addition to GT and clustering of unidirectional genes, consistent with other dinoflagellate genomes [4, 5, 25] (Fig. 1c, d). This genome included ~ 30% repetitive elements composed of simple repeats (1.97%), low complexity repeats (0.39%), satellite repeats (0.02%), LINEs (0.02%), LTR elements (0.03%), DNA elements (0.1%), and unclassified repeats (27.4%) (Additional file 2: Supplementary Tables 3 and 4). The abundance of repetitive elements may drive genome evolution in dinoflagellates, as reported in Symbiodiniaceae and *Polarella glacialis* genomes (16–68%) [6, 7]. Comparative analysis of intron and exon features of *A. gibbosum* provides additional insights into expansion of dinoflagellate genomes (Table 1). Intronic length in *A. gibbosum* genome is ~ 1.7 Gb, so the intronic region accounts for ~ 27% of the genome, whereas in the Symbiodiniaceae and *Polarella glacialis* genomes, the average total intronic lengths are 411.5 kb and 737.1 kb, respectively. Despite average exon lengths ranging from 99 to 185 bp, *A. gibbosum* has the lowest dinoflagellate exon density, with 8.1 exons per gene, compared with 11.3–19.6 exons per gene for other species (Table 1). Large introns have several biological implications, including high energy requirements during transcription, delays in protein production, and greater potential for errors in intron splicing [26, 27]. It follows that some advantage must compensate for such long introns.

**Table 1:** Statistics of the *A. gibbosum* genome assembly and those of some available dinoflagellate genome assemblies

		***Amphidinium gibbosum***	***Breviolum minutum*** (B1) [4]	***Symbiodinium tridacnidorum*** (A3) [9]	***Cladocopium*** sp***.*** (C) [9]	***P. glacialis*** CCMP1383 [6]	***P. glacialis*** CCMP2088 [6]
	Total assembly length (bp)	7,034,147,423	615,520,517	766,659,703	704,779,698	2,984,680,192	2,756,104,381
	N50 of scaffold (bp)	166.4k	126.2k	133.4k	248.9k	170.3k	129.2k
	G+C content (%)	47.1	43.6	49.9	43.0	45.9	46.2
**Genes**	No. of genes	85,139	41,925	69,018	65,832	58,232	51,713
	Average length of genes (bp)	26,201	11,959	8834	8192	16,206	13,931
	Average length of transcripts (nt)	1423	2067	1423	1479	1230	1178
	Gene models supported by EST (%)	85.2	77.2	67.5	62.5	94.0	94.3
**Exons**	No. of exons per gene	8.1	19.6	13.38	11.3	11.6	10.8
	Average length (bp)	185	99.8	105	130	105.7	108.7
	Total length (Mb)	126	82.1	98.2	97.3	71.6	60.9
**Introns**	No. of genes with introns (%)	93.7	95.3	83.4	80.3	73.8	75.6
	Average length (bp)	3468	499	561	622	1408.0	1296.0
	First two nucleotides at 5′ splice sites	GT/GC/GA	GT/GC/GA	GT/GC/GA	GT/GC/GA	GT/GC/GA	GT/GC/GA
	Total length (Mb)	1715	331.5	481.8	421.2	838.0	636.2

To understand whether *A. gibbosum* gene models are conserved at the pathway level, predicted genes were mapped to KEGG reference pathways and compared with those of other dinoflagellates and eukaryotes. This resulted in the recovery of 388 KEGG pathways, indicating that the *A. gibbosum* genome has most of the pathways present in other eukaryotes (Fig. 1e). Pfam analysis showed Leucine-rich repeat (LRR), Ankyrin, Tetratricopeptide (TPR), and Pentatricopeptide repeat (PPR) domains as the most abundant domains in *A. gibbosum* (Additional file 2: Supplementary Table 2). Compared with eukaryotes, these repeat domain families, which often contribute to duplication events and to protein-protein interactions, are more abundant in dinoflagellates [9, 28].

### Diversified roles of NRPS adenylation domains in dinoflagellates

**Fig. 2:**
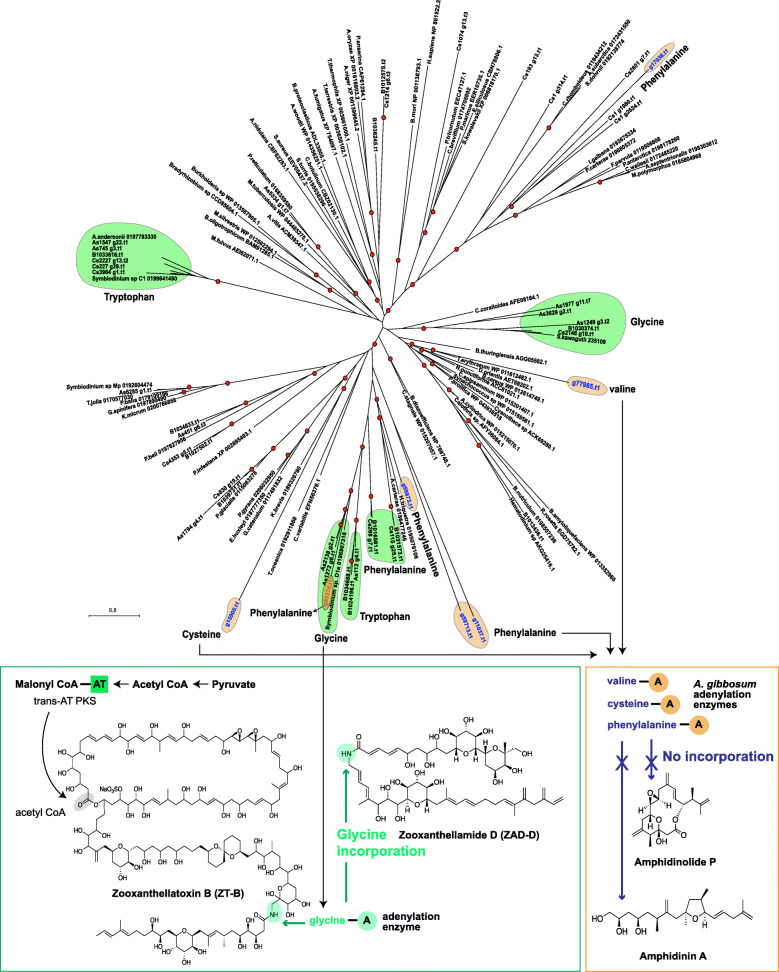
Affinities of adenylation domains from dinoflagellates show the importance of glycine as a substrate for biosynthesis of specialized toxin secondary metabolites. A molecular phylogenetic tree of adenylation domains indicates protein diversification in Symbiodiniaceae and *A. gibbosum*. Green- and orange-shaded regions indicate adenylation-domain affinities in Symbiodiniaceae and *A. gibbosum,* respectively. The Symbiodiniaceae can incorporate glycine (green box) during specialized toxin secondary metabolite biosynthesis such zooxanthellatoxin B (ZT-B) and zooxanthellamide D (ZAD-D), whereas *A. gibbosum* does not utilize glycine, yielding the simple nitrogen-lacking polyketides, amphidinin A and amphidinolide P. *A. gibbosum* adenylation sequences are denoted in blue. Red dots indicate a posterior probability ≥ 0.75 using Bayesian inference

In order to understand evolution and functions of secondary metabolite genes in *A. gibbosum*, we conducted molecular phylogenetic analyses of the *PKS* and *NRPS* gene families. This confirmed the extensive diversification of these enzyme genes, as previously reported (Fig. 2 and Additional file 1: Supplementary Fig. 2) [10]. Detailed analysis of the adenylation (A) domain of NRPS revealed how specialized metabolites arise in dinoflagellates. The NRPS adenylation domain is the first enzyme in the NRPS complex that selectively incorporates amino acids into NRPSs for biosynthesis of peptide-based natural products, as well as hybrid PKS/NRPS metabolites [11]. The adenylation (A) domain can function as a freestanding protein (Additional file 1: Supplementary Fig. 3), a clear deviation from the usual assembly-line enzymology, known in bacterial genomes [29]. We found that freestanding A domains in *A. gibbosum* utilize cysteine, valine, and phenylalanine as substrates (Fig. 2), instead of glycine, tryptophan, and phenylalanine, the main substrates utilized by the Symbiodiniaceae [10].

Glycine is incorporated into complex metabolites in the Symbiodiniaceae by bridging and forming hybrid molecules, such as zooxanthellatoxin B (ZT-B) and zooxanthellamide D (ZAD-D) [30, 31]; however, none of the amphidinolides and related polyketides [17, 32] isolated from *A. gibbosum* (amphidinin A and amphidinolide P) contains glycine, resulting in smaller, simpler molecules. Marine dinoflagellates synthesize polyketides that are usually polyol in nature [33]. The carbon skeleton of these polyketides is commonly assembled from acetate, with the rare addition of glycine to form hybrid polyketides [34]. Glycine remains the only amino acid substrate reported in metabolites isolated from dinoflagellates [35, 36], and our analysis suggests that the unique substrate affinities of the NRPS adenylation domain contribute to metabolite complexity in dinoflagellates.

### Secondary metabolite biosynthesis responses depend on nutrient starvation regimes

**Fig. 3:**
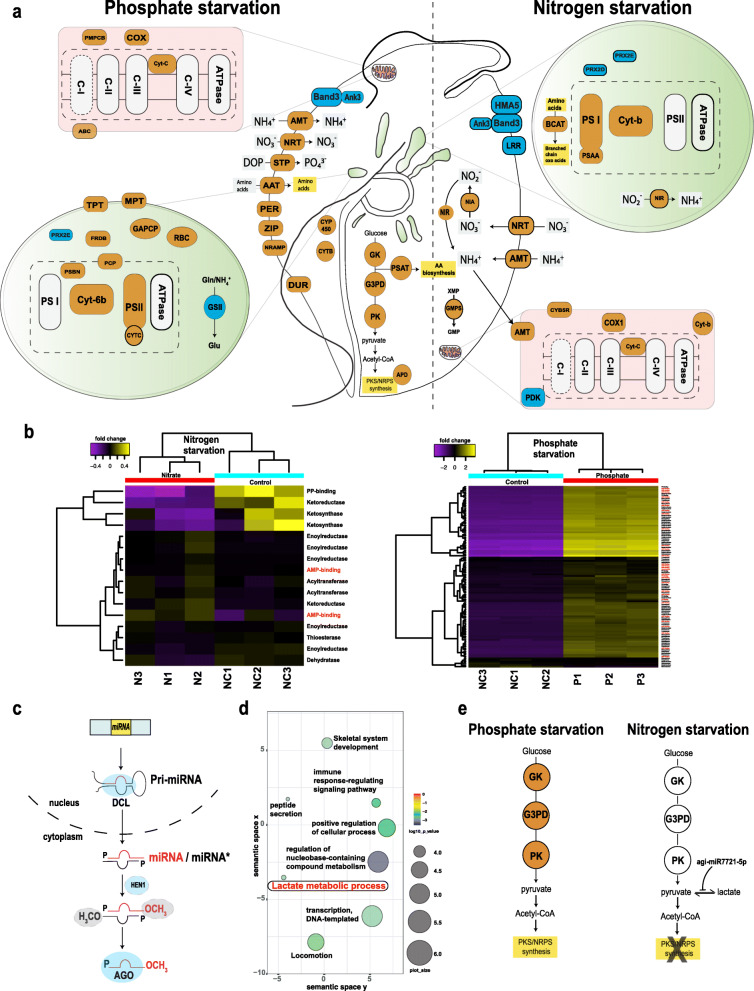
Differentially expressed genes (mRNAs and microRNAs) during nitrogen and phosphate starvation in *Amphidinium gibbosum*. **a** Schematic cellular overview of the main differentially expressed genes during nitrogen and phosphate starvation. Orange and blue coloring indicate up- and downregulation, respectively. Green ovals represent plastids, and red boxes indicate mitochondria. A detailed description of proteins is given in Additional file 2: Supplementary Table 9. **b** Expression profile of *PKS* and *NRPS* genes (*q* < 0.05 and |log2(FC)| > 2) under nitrogen and phosphate starvation. Values show fold changes while N1, N2, and N3; P1, P2, and P3; and NC1, NC2, and NC3 denote triplicate nitrogen, phosphate, and control samples, respectively. Details of the genes are provided in Additional file 2: Supplementary Table 10. *NRPS* and *PKS* genes are denoted in red and black, respectively, along the *y*-axis. **c** The presence of Dicer (DCL), HEN1, and AGO proteins indicates functional RNAi machinery in *A. gibbosum*, supported by genomic and transcriptomic data. Whether mature miRNAs in *A. gibbosum* are methylated is unknown (shaded gray). **d** Enrichment of miRNA targets during nitrogen starvation shows lactate metabolism as an enriched target process. **e** The miRNA, agi-miR7721-5p, targets pyruvate metabolism under nitrogen starvation, affecting secondary metabolite biosynthesis. Orange coloring indicates upregulation

Several studies have demonstrated that nitrogen and phosphorus sources and their availabilities impact both biomass and secondary metabolite production in marine organisms [20–22]. It remains unclear which nutrient combinations or limitations drive toxin formation, and this motivated us to investigate whether nutrient starvation affects secondary metabolism in *A. gibbosum.* We performed deep transcriptome sequencing, recovering 422 pathways, with “metabolic pathways” and “biosynthesis of secondary metabolites” accounting for 1187 proteins (Additional file 1: Supplementary Fig. 1f and Additional file 2: Supplementary Table 5). Under nitrogen starvation, only 16 secondary metabolism genes (*PKS* and *NRPS*) were differentially expressed (|log2(FC)| > 2, *q* < 0.05) (Fig. 3a, b). Gene ontology (GO) enrichment showed that nitrogen starvation has significant effects on nitrogen transport and metabolism (*AMT*, *NRT*, *NIA*, and *NRT* genes were upregulated) and on anion export (*Band* 3 gene was downregulated) (|log2(FC)| > 1, *p* < 0.001) (Fig. 3a and Additional file 1: Supplementary Fig. 4a, b). KEGG pathway enrichment confirmed nitrogen metabolism as the most enriched pathway among upregulated genes, while pathways related to bicarbonate release were the most downregulated genes (*p* < 0.001) (Additional file 2: Supplementary Table 6). Our analysis revealed novel details about gene expression changes under nitrogen starvation [37]. *A. gibbosum* apparently tunes its carbon level and nitrogen intake during starvation by downregulating the bicarbonate export system (*Band* 3 gene) (Fig. 3a). Overall, our data indicate that *A. gibbosum* modulates incorporation and utilization of several forms of dissolved organic and inorganic nitrogen to respond to nitrogen availability.

Under phosphate starvation, however, 108 *PKS* and *NRPS* unigenes were differentially expressed at |log2(FC)| > 2 and *q* < 0.05 (Fig. 3b and Additional file 1: Supplementary Fig. 5a). Gene ontology (GO) enrichment showed that phosphate starvation upregulates small molecule biosynthesis and downregulates anion release (|log2(FC)| > 2, *p* value < 0.001) (Fig. 3a and Additional file 2: Supplementary Table 6b). KEGG pathway enrichment confirmed that ribosome, metabolic pathways, and biosynthesis of secondary metabolite pathways are the most enriched pathways among upregulated genes (*p* < 0.001) (Additional file 2: Supplementary Table 6). During phosphate starvation, membrane transporters (STP, ZIP, AMT, NRT, and AAT) involved in uptake of amino acids, ammonium, dissolved organic phosphate (DOP), metal ions, and nitrate were significantly upregulated. Insufficient dissolved inorganic phosphate can be overcome by utilizing DOPs, which are hydrolysed to release phosphate [38]. This suggests that *A. gibbosum* can utilize various sources of phosphorus while downregulating genes involved in bicarbonate export, similar to the response observed during nitrogen starvation. Key components of the ATP-consuming glycolytic pathway (e.g., glucokinase, glyceraldehyde-3-phosphate dehydrogenase, and pyruvate kinase) and several ribosomal proteins were significantly upregulated since they are involved in ATP-driven protein synthesis to meet cellular demand for metabolism and phosphate uptake. In both starvation treatments, hierarchical clustering of *NRPS* and *PKS* gene expression values revealed two main clusters (Fig. 3b), indicative of a set of co-expressed genes needed for secondary metabolite biosynthesis.

Dinoflagellate carbon-fixing potential increased during phosphate starvation, with several key plastid components (Fig. 3a) being upregulated, including phosphate transporters. This increase may be necessary to fuel augmented cellular processes, as observed in the alga, *Prymnesium parvum* [39]*.* Dinoflagellate toxin production changes when environmental parameters such as light, temperature, salinity, and nutrient levels shift [40]. The present analysis shows that the *PKS* and *NRPS* genes are upregulated when dinoflagellates are subjected to phosphorus starvation (Fig. 3b) and this can be explained evolutionarily, where microalgal growth slows under nutrient limitation, as cells divert carbon resources for defense [41] (Fig. 3a). Consistent with this theory, increased photosynthetic activity observed during phosphorus starvation in *A. gibbosum* would be a coordinated physiological response to provide energy necessary for secondary metabolite biosynthesis.

### Possible regulation of toxin biosynthesis by microRNAs during nutrient starvation

Based on the low expression of *PKS* and *NRPS* unigenes under nitrogen starvation (Fig. 3b), we questioned whether post-transcriptional regulation by microRNAs could be involved. We found expected components of RNAi machinery in *A. gibbosum* consistent with previous reports [7, 42–45] (Fig. 3c and Additional file 1: Supplementary Fig. 6). Using the sequenced genome and expressed small RNA data, under phosphate starvation, we found that two miRNAs (agi-miR-6874-5p-2 and a new miRNA denoted, aginovel-mir-0021) were differentially expressed (*q* value < 0.05, log2(FC) > 2). Upregulation of the two miRNAs was > 18× compared to the control, suggesting that they could have significant effects during phosphate starvation. Indeed, under phosphate starvation, the two upregulated miRNAs targeted pathways involved in fructose-mannose metabolism, proteoglycan synthesis and N-glycan biosynthesis (enrichment > 4×, *p* < 0.01, Fisher’s exact test) (Additional file 2: Supplementary Table 7). Under nitrogen starvation, we found one miRNA (agi-miR7721-5p) that was differentially expressed (*q* value < 0.05, log2(FC) > 2). *Amphidinium gibbosum* had 303 potential target genes, and KEGG pathway target enrichment identified pyruvate-lactate metabolism as a major target (38.4× enrichment, *p* < 0.001, Fisher’s exact test) (Fig. 3d, e, Additional file 1: Supplementary Fig. 7, and Additional file 2: Supplementary Table 7). This would directly affect production of acetyl-CoA, which is synthesized from pyruvate, a key substrate for polyketide biosynthesis [46], thereby regulating secondary metabolism. No significant *PKS* and *NRPS* gene upregulation was observed under nitrogen starvation, in which miRNA-mediated post-transcriptional regulation might affect secondary metabolism by targeting pyruvate biosynthesis. miRNA effects on secondary metabolite biosynthesis have been reported in plants [47, 48].

### Transcriptome sequencing reveals diversity of PKS transcripts

**Fig. 4:**
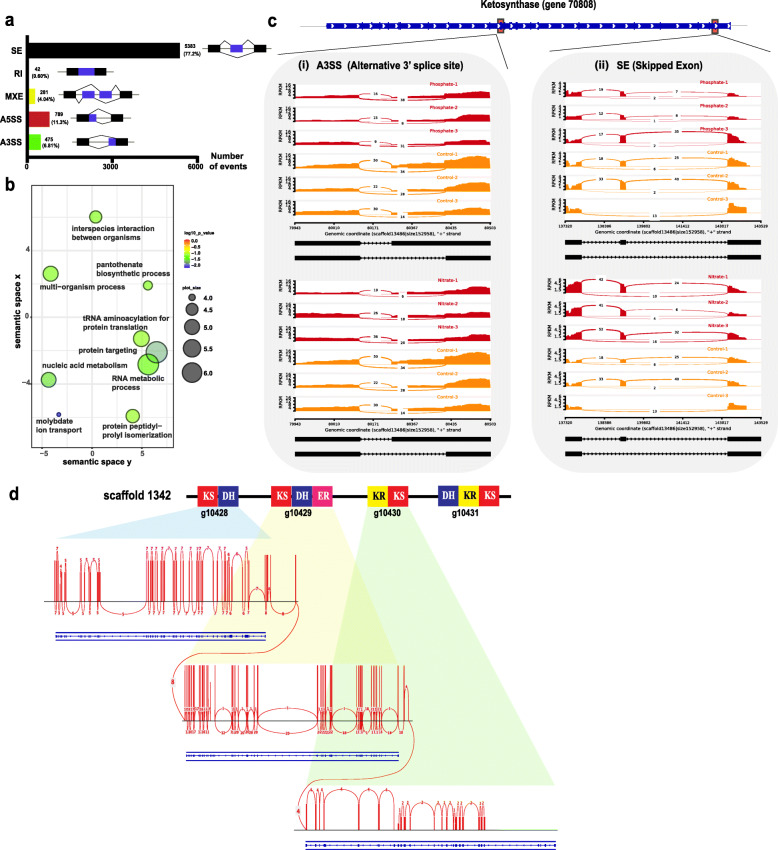
Alternatively spliced isoforms and polycistronic *PKS* gene expression in *Amphidinium gibbosum*. **a** AS events and their frequencies. SE “skipped exon,” RI “retained intron,” MXE “mutually exclusive exon,” and A3SS and A5SS “alternative 3′ and 5′ splice events”. Black boxes indicate constitutively spliced exons while blue boxes represent alternatively spliced exons. **b** Gene ontology (GO) biological processes showing significant enrichment of all genes undergoing alternative splicing. **c** Alternative 3′ splice sites (i) and skipped exons (ii) were identified on a ketosynthase gene (g70808) on scaffold 13486. Phosphate and nitrate experiments are shown in red while controls are in orange. Expression is plotted on the *y*-axis, genomic coordinates on the *x*-axis, and isoforms are at bottom in black, with exons depicted in black boxes. Read coverage is represented with numbers. **d** Sashimi plot showing three uni-directionally aligned *PKS* genes on scaffold1342 (colored in blue) with multiple polycistronic transcripts (red lines) spanning these genes. PKS module organization within genes is based on PFAM annotation. Iso-Seq read coverage is represented by red vertical blocks, and splicing junction support is shown with numbers. Exons are shown in blue blocks, and lines between blue blocks represent introns. KS "ketosynthase," DH "dehydratase," ER "enoylreductase," KR "ketoreductase"

Alternative splicing (AS) is an important post-transcriptional regulatory mechanism, whereby a single gene can generate multiple mRNAs, increasing their diversity and complexity [49]. We surveyed five major AS types using rMATS [50] and identified 6970 AS events across 5417 genes, with skipped exons (SE) being the most common AS event (77.2%) (Fig. 4a), followed by alternative 3′splice sites (A3SS) and alternative 5′splice sites (A5SS) (6.8% and 11.3%, respectively). In order to determine biological processes of genes associated with alternative splicing, identified by rMATS [50], GO enrichment was performed. This revealed that ion transport, nucleic acid metabolism, and RNA metabolic process are the most enriched terms (Fig. 4b). Subsequently, we assessed whether AS events were associated with *PKS* genes. AS landscape analysis at the genome-wide level revealed one *PKS* gene (g70808) that underwent two AS events, A3SS and SE (Fig. 4c, Additional file 1: Supplementary Fig. 8a). With differential exon usage (DEU) analysis, we found 1 exon (E026) that was differentially expressed (*q* value < 0.05) during nitrogen starvation (Additional file 1: Supplementary Fig. 8b). AS events function in plant growth and stress responses [51]. Proteins resulting from differently spliced isoforms of the same gene can have different subcellular localization and can inhibit formation of alternative homo- and hetero-dimers [52, 53].

To understand how splice junctions contribute to multifunctional polyketide synthase (PKS) isoforms, we conducted Pacbio Isoform sequencing and recovered several transcripts that contained all PKS domains except the acyltransferase (AT) domain, suggesting the *trans*-acting nature of these enzymes (Additional file 1: Supplementary Fig. 8c). *AT* genes were indeed *trans*-acting and belong mainly to the family of malonyl-CoA ACP transferase, contributing malonyl-CoA for chain elongation (Additional file 1: Supplementary Fig. 2b). By mapping these isoforms on the *Amphidinium* genome, we identified *PKS* polycistronic transcripts span multiple genes (Fig. 4d). Based on the presence of multiple *PKS* genes in the genome and their predicted signal peptides (Additional file 1: Supplementary Fig. 2), we asked whether these proteins are localized within the cell. Immunolocalization of ketosynthase and ketoreductase proteins showed that they are localized in mitochondria, chloroplasts, and secretory bodies, as previously reported (Additional file 1: Supplementary Fig. 9) [54]. Additionally, we detected PKS proteins in membrane vesicles, suggesting possible new functions, as demonstrated by their facilitation of nucleation in otolith mineralization [55]. Further functional studies of these proteins will be revealing. By combining different sequencing technologies, we detected polycistronic PKS transcripts, as well as AS events in *PKS* genes, deepening our understanding of dinoflagellate secondary metabolism. Based on long Iso-Seq reads, we investigated whether secondary metabolite biosynthetic genes contain spliced leader (SL) sequences at their 5′ ends. In dinoflagellates, mRNA maturation is thought to require trans-splicing of the SL sequence [56]. We recovered 548 sequences containing the SL and the relict SL signature, but no *PKS* transcripts contained it. This could be due to transcript degradation or to a lack of SL sequences at 5′ ends of these transcripts.

### Iterative secondary metabolite biosynthesis in dinoflagellates

**Fig. 5:**
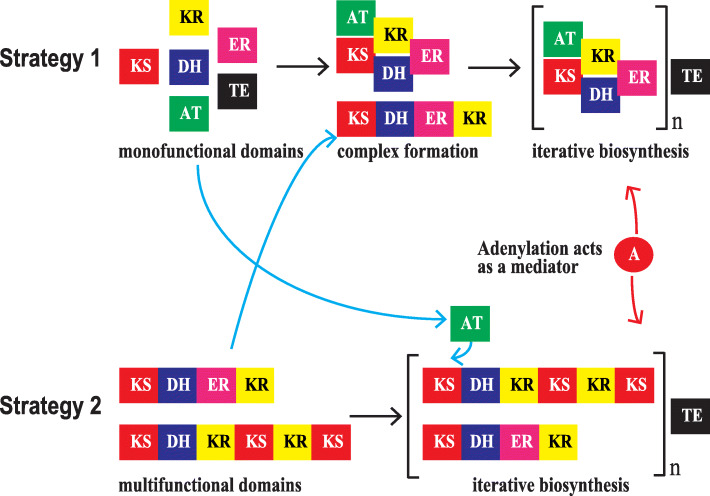
Strategies for secondary metabolism in dinoflagellates based on a genomic survey. Acetyltransferase acts *in trans* to provide activated substrates to acyl carrier protein (ACP) with extensions and modifications by optional domains, terminating with hydrolysis by thioesterase. The adenylation domain activates the amino acyl substrate and bridges intermediate products, acting as a mediator. KS, ketosynthase; KR, ketoreductase, AT, acetyltransferase; DH, dehydratase, ER, enoylreductase; TE, thioesterase; A, adenylation. ACP are omitted for clarity

Polyketide biosynthesis resembles that of fatty acids. The chain is initiated with acetyl-CoA, extended in a series of Claisen ester condensation reactions with malonyl-CoA, and terminated when the required length is reached [10]. While amphidinolides are unique in structure and bioactivity, some similarities exist among them [17], suggesting a common biogenic origin. Complete biosynthesis of an amphidinolide would require all genes present in a cluster, representing up to 500 kb of genomic DNA [11, 18]. Our genomic survey of *A. gibbosum* confirmed that such long clusters of *PKS* genes are not present. Each ketosynthase enzyme contributes two carbons to a growing polyketide chain, so a 26-membered polyketide would require at least twelve rounds of carbon addition, implying that such a long cluster is not present in *A. gibbosum*. Thus, secondary metabolite biosynthesis in dinoflagellates can occur in two ways: (1) monofunctional, separate PKS proteins form an enzyme complex and iteratively catalyze addition of substrate, or (2) multifunctional small PKS proteins utilize substrate in many cycles, to yield a product stabilized by repeat domains that assist such protein-protein interactions (Fig. 5) [57–59]. Both these strategies resemble the iterative mono- and multifunctional PKSs of bacterial and fungal systems [60, 61], acquired by horizontal gene transfer [10]. Cross talk between these two co-occurring strategies in dinoflagellates could be mediated by the *trans*-acting acyltransferase (AT) and NRPS domains, considering that sets of secondary metabolic genes tend to be co-expressed during metabolite biosynthesis (Fig. 3b).

## Conclusions

In this study, we applied an integrated omics approach to understand dinoflagellate secondary metabolite biosynthesis. To this end, we sequenced the genome of *A. gibbosum* and identified key features that regulate secondary metabolite levels and structural diversity. We hypothesize that miRNA-mediated, post-transcriptional regulation in *A. gibbosum*, which targets primary pyruvate metabolism, subsequently affects secondary metabolism. This study represents a first step to illuminate key molecular events involved in dinoflagellate secondary metabolism, and it should facilitate studies of HAB formation and associated toxin production. Ongoing high-throughput sequencing of dinoflagellate genomes promises to be informative, not only for understanding toxin secondary metabolism genes, but also for better insights into their genome organization. The availability of this first basal dinoflagellate genome provides important clues about dinoflagellate evolution and extends the genome size limit that has been a challenge for several years.

## Methods

### Biological sample

*Amphidinium gibbosum* was isolated from inner cells of a marine acoelomorph, *Amphiscolops* sp., collected near Ishigaki Island, Japan. The culture was maintained in artificial seawater (ASW) containing 1X Guillard’s (F/2) marine-water enrichment solution and an antibiotic-antimycotic mix in a 25 °C incubator under a 12:12 light and dark cycle. Subculture was performed with fresh medium approximately every 4 weeks and was handled aseptically. For transmission electron microscopy (TEM), cells were fixed in 2.5% glutaraldehyde for 1 h, washed 3× with 0.1 M cacodylate buffer, and incubated in 1% osmium tetroxide for 30 min. Cells were then washed and dehydrated in an ethanol series (70%, 80%,90%, 95%, 100%, 100%, 100%), at 5-min intervals. Samples were infiltrated with ethanol-Epon resin for 30 min and steeped in 100% resin overnight. The resin was polymerized at 60 °C for 2 days. Sections were cut using a diamond knife and viewed under a JEM-1230R JEOL microscope. The phylogenetic position of *A. gibbosum* was confirmed by aligning and trimming partial LSU rDNA sequences of several dinoflagellates and performing maximum likelihood analysis using RaxML [62]. Phylogenetic assignment was consistent with the taxonomic description [63].

### Genome size estimation

For *A. gibbosum* genome size estimation, nuclear DNA from three replicates was measured using fluorescence-activated cell sorting (FACS) with *Xenopus laevis* (*n* = 3) as an internal control of known genome size. Nuclear extraction and staining were performed using a Partec CyStainPI absolute T kit (Partec #05-5023), following the manufacturer’s protocol, and fluorescence signals were measured with a BD Accuri C6 cell analyzer (BD Bioscience). The reported measurement for *A. gibbosum* reflects the 1C genome content, as *Amphidinium* is reportedly haploid in culture. *K*-mer analysis was performed using Jellyfish (v2.1.3) [64], and resulting histograms were visualized using GenomeScope [65] to survey the genome size and repeat content.

### DNA sample preparation and sequencing

Cells were centrifuged at 3000*g* for 10 min and washed using TEN buffer (100 mM Tris-Cl pH 8, 100 mM EDTA pH 8, 1.5 M NaCl, 0.5 mg/mL proteinase K, and 7% SDS) for 2 h at 65 °C so as to lyse bacterial contaminants. DNA was extracted using a modified protocol [66] of gentle rotation for 1 h after addition of chloroform-isoamyl alcohol (24:1) before ethanol precipitation [4]. Isolated DNA was further cleaned using ethanol precipitation. DNA was fragmented and paired-end libraries with an insert size of 620–820 bp were prepared. Libraries were quantified by qPCR and sequenced using an Illumina Miseq, according to the manufacturer’s protocols. This generated ~ 10 Gb of 2 × 300 bp paired-end data. The same library was further sequenced using a Hiseq 2500, generating ~ 586 Gb of 2 × 125 bp of data. Reads were merged and trimmed using Trimmomatic (v0.35) [67] and were quality-checked using FastQC (v0.11.4) [68]. Additionally, 12 mate-pair libraries were constructed using Nextera technology with 2–18-kb inserts selected using the Bluepippin and SageELF systems. Mate-pair libraries were sequenced with a Hiseq 4000, generating ~ 200 Gb of data. Raw mate-paired reads were filtered using NextClip (v1.31) [69]. Genome assembly employed Platanus (v2.1.4) [70], and the assembled genome was subjected to two rounds of scaffolding with SSPACE (V3.0) [71]. Gaps in scaffolds were filled using GapCloser (v1.12) [72] (Additional file 1: Supplementary Fig. 10A).

### Evaluation of genome assembly completeness and removal of contaminating sequences

The scaffolded *Amphidinium* genome was checked for genome completeness using BUSCO 303 highly conserved eukaryotic genes (CEGs) [73]. Additionally, the BLAST suite was used to recover 458 CEGs from CEGMA [74] against the *Amphidinium* genome to identify potential homologs at a cutoff value of 1e^−5^. To identify bacterial and viral contaminants, we conducted a BLASTN search against several databases that we built by retrieving draft and complete bacterial genomes and viral genomes from NCBI and PhanToME. A combination of cutoffs (total bit score > 1000, E ≤ 10^−20^) was used to identify scaffolds with similarities to bacterial and viral sequences.

### cDNA construction, Iso-Seq sequencing, and data processing

RNA was extracted from cells growing under standard conditions (12:12 light and dark cycle), and a cDNA library was constructed using a TruSeq Stranded RNA Sample Prep Kit (Illumina). Libraries were quantified and validated by qPCR and with a 2100 Agilent Bioanalyzer, respectively. The validated library was subsequently sequenced using two lanes of Hiseq 2500 (Illumina). Reads were trimmed using Trimmomatic (v0.35) [67], quality-checked using FastQC (v0.11.4) [68], and assembled de novo using Trinity (v2.3.2) [75]. For Iso-Seq sequencing, RNA was extracted from several culture treatments and pooled. High-quality RNAs (RIN > 7.0) were used for cDNA synthesis using a Clontech SMARTer PCR cDNA kit. Size fractionation (0.7–2.5, 2.5–7, and > 7 kb) was conducted using the SageELF system (Sage Science, Beverly, MA, USA). Libraries were sequenced with the Pacific Biosciences RS II platform (P6-P4 chemistry) and a 360-min movie length. In total, 16 SMRT cells were sequenced. Raw sequencing data were processed using the RS_Iso-Seq protocol. HQ and LQ reads were error-corrected by employing proovread (v2.14) [76] using Illumina RNA-seq data. Reads were then merged, and “cd-hit-est” from CD-HIT (v4.6) [77] was used to remove redundancy with parameters: -c 0.99 -G 0 -aL 0.00 -aS 0.99 -AS 30 -M 0 -d 0 -p 1 -T 24. Non-redundant transcripts were further processed with Cogent (https://github.com/Magdoll/Cogent). Polished Iso-Seq sequences were surveyed for the dinoflagellate spliced leader (CCGTAGCCATTTTGGCTCAAG) and the relict dinoSL (CCGTAGCCATTTTGGCTCAAGCCATTTTGGCTCAAG) [78] sequences using BLAST with no gaps and up to 1 mismatch permitted.

### Repetitive element annotation and gene model prediction

In order to confirm splice sites, the assembled transcriptome was mapped to the assembled genome using GMAP [79]. For annotating transposable elements (TEs), de novo repeats within the genome were identified using an l-mer size of 17 bp with RepeatScout [80]. A combined library was made, consisting of de novo repeats and eukaryotic TEs from RepBase. This library was then used to locate and annotate repetitive elements in the assembled genome using RepeatMasker [81]. RNA-seq reads were mapped to a soft-masked genome using STAR [82] and the BRAKER2 pipeline [83]. UTR and gene model prediction were performed with Augustus (v3.2.3) [84]. To improve gene prediction accuracy, intron and exon hints were generated as additional evidence of gene structure and location by mapping Illumina and Iso-Seq transcripts to the genome with GMAP [79] and STAR [82]. Hints were then used to perform final gene prediction using a modified version of Augustus (v3.2.3) [84], in which the source code was changed in consideration of non-canonical exon-intron boundaries. The final set of predicted proteins was annotated against UniProt [85] and PFAM [86]. Briefly, BLASTP searches for all protein models were performed with the SwissProt and TrEMBL databases (October 2018 release). Amino acid sequences were subjected to PFAM [86] domain searches using HMMER (v3.1b2) [87], and hits larger than 1^e−5^ were discarded. For KEGG pathway analysis, the online service on the KEGG Automatic Server (KAAS) was used to assign predicted genes to KEGG orthologs (bi-directional best hit method) and mapped orthologs to KEGG pathways.

### Phylogenetic analysis of PKS and NRPS proteins and prediction of substrate specificities

The dataset used previously [10] was repopulated with ketosynthase, acyltransferase, adenylation, and condensation protein sequences from the *A. gibbosum* genome. Briefly, four datasets were created, consisting of 244 KS sequences (225 aa), 104 AT sequences (208 aa), 121 A-sequences (272 aa), and 111 C-sequences (253 aa). Mono- and multifunctional domain-containing sequences were aligned using MUSCLE [88], and domain areas with best alignment were retained while regions with ambiguity were removed. Two methods for phylogenetic reconstruction were used, maximum likelihood using RaxML [62] (1000 bootstraps and LG + G model) and Bayesian inference (run to a maximum of 6 million generations plus 4 chains, or until probability approached 0.01), using MrBayes (v3.2) [89]. Substrate specificity of *A. gibbosum* AT sequences was generated using I-TASSER [90]. In order to determine the A-domain specificity and C-domain types, the LSI-based A-domain predictor and NaPDos were used, respectively [91, 92]. The phylogenetic analysis of A-domain and a part of its substrate specificity are depicted in Fig. 2. Sequence alignment of the A-domain is provided as Additional File 3. PKS protein subcellular localization was detected using ChloroP 1.1 and TargetP 1.1 and was further confirmed with DeepLoc [93–95].

### Nutrient starvation experiment

For a nitrate-starved culture, the culture medium was prepared by supplementing artificial seawater (ASW) with F/2 medium containing a reduced nitrate concentration (150 μM). For a phosphate-starved culture, the phosphate level was 22 μM. A phosphate and nitrate-replete treatment was set up as the control, in which nitrate and phosphate concentrations were 880 and 36 μM, respectively. Both starvation (depleted) and control treatments were conducted in triplicate (*n* = 3). First, measurements were started after 24 h of stabilization, and this was counted as day 1. Nitrate and phosphate levels were monitored using the Griess and phosphomolybdenum blue spectrophotometric methods, respectively [96, 97], until their concentrations were undetectable. Other physiological parameters, such as cell concentration, chlorophyll *a*, and photochemical efficiency (*F*v/*F*m ratio), were also monitored (Additional File 1: Supplementary Fig. 10B). Cell counts were obtained by fixing cells in formalin and using a hemocytometer for visualization. 1-mL samples were centrifuged, and cell pellets were immersed in *N*,*N*-dimethylformamide (DMF) and kept at − 20 °C for at least 12 h in order to extract chlorophyll *a*, which was measured using a Turner Trilogy (Turner Designs fluorometer, USA) and then averaged to content per cell. Photochemical efficiency was monitored with a Xe-PAM (Walz, Germany).

### Gene expression analysis during nutrient starvation

When dissolved nitrate and phosphate reached an undetectable level, ~ 10^7^ cells were collected, snap frozen in liquid nitrogen, and ground using a cryopress. RNA was extracted from 3 control, 3 nitrate-starved, and 3 phosphate-starved samples using PureLink reagent. Four micrograms of RNA was used for cDNA library construction with a TruSeq Stranded RNA Sample Prep Kit (Illumina). Libraries were quantified and validated by qPCR and with a 2100 Agilent Bioanalyzer, respectively, and sequenced in two lanes of a Hiseq 4000 (Illumina). Reads were trimmed using Trimmomatic (v0.35) [67], quality-checked using FastQC (v0.11.4) [68], and assembled using Trinity (v2.3.2) [75]. The assembly was processed with CD-HIT-EST (v4.6.7) [77] using a clustering threshold of 0.95. Functional annotation of non-redundant contigs was performed using BLAST with several databases: UniProt, GeneBank non-redundant (nr), Kyoto Encyclopedia of Genes and Genomes (KEGG), and eggNOG (*E* value cutoff of 10^−5^) [85, 98]. Transcriptomic gene completeness was evaluated using BUSCO (v3.0.2) [73]. For identification of differentially expressed transcripts, expression abundance was quantified using RSEM [99]. The R package, EdgeR [100], was used to identify differentially expressed genes with adjusted *p* values (*q* value) determined with the Benjamini, Krieger, and Yekutieli correction of the PRISM package. Figure 3a, b depicts the results of this analysis. Gene ontology term functional enrichment was performed using Fisher’s exact test in topGo with the parent-child analysis to categorize whether differentially expressed genes were enriched in molecular function, cellular components, and biological processes [101]. KEGG pathway enrichment was performed using DAVID [102] by applying Fisher’s exact test.

### Small RNA sequencing for the nutrient starvation experiment

Small RNAs were isolated from the same RNA pellet (*n* = 3) collected from the depleted-replete experiments using the NEXTflexTM Small RNA-seq Kit V3 (Bioo Scientific). Single-end reads (1 × 50 bp) were generated on a Hiseq 2500 platform. Reads were cleaned by removing adapter and polyA/N sequences using Cutadapt-1.4.1 [103], and reads within the range of 17–25 were retained. Reads were further collapsed using the collapse_reads.pl script of the MiRDeep2 package [104]. Sequences having hits to various non-coding RNAs (rRNAs, tRNAs, snRNAs, snoRNAs, and scRNAs) of the RNAcentral database (The RNAcentral Consortium, 2015) were discarded. Bowtie (v1.1.12) [105] was used to map clean, small RNA reads to the *Amphidinium gibbosum* genome with no mismatches and 1 alignment. Mapped reads were further queried against known miRNAs in miRBase 22.0 (http://www.mirbase.org). miRNAs were annotated using the miRdeep2 package. Previous miRNA criteria [42] were applied to the list of annotated miRNAs. miRNA expression level profiling was conducted and normalized using the quantifier.pl script of the miRdeep2 package where processed reads were mapped to identified miRNA precursors. EdgeR [100] was then used to identify differentially expressed miRNAs at FDR < 0.05 (adjusted *p* value), as determined by Benjamini, Krieger, and Yekutieli of the PRISM package and |log2(FC)| > 1. Only miRNAs present in at least 2 replicates were considered further. For predicting mRNA targets of the miRNAs, 3′UTR sequences of unigenes were used by miRanda [106] under strict criteria. GO and KEGG pathway enrichment was performed for predicted target unigenes of differentially expressed miRNAs using topGO and DAVID, respectively [101, 102]. Figure 3c–e depicts the results of this analysis.

### Identification of key proteins in microRNA biogenesis pathways

In order to confirm the presence of a miRNA biogenesis pathway, sequences of three core protein families involved in RNA interference (i.e., Argonaute, Dicer, and HEN1) were retrieved for model organisms (*H. sapiens*, *C. elegans*, *S. pombe*, *D. melanogaster*, and *A. thaliana*) from UniProtKB [85]. Sequences were then queried against predicted proteins from the *A*. *gibbosum* transcriptome using BLASTP (*E* value cutoff of 10^−10^). Hits were then searched for specific domains (a PAZ domain and a pair of RNase III domains for Dicer, Piwi and Dicer domains for Argonaute, and a methyltransferase domain for HEN1) needed for functional activity using InterProScan [107]. Alignment of homologs against retrieved RNAi proteins from model organisms was conducted using Clustal Omega [108] and visualized using Jalview [109].

### Alternative splicing (AS) and enrichment analyses

In order to identify alternative splicing events (Skipped exon [SE], alternative 5′ splice site [A5SS], alternative 3′ splice site [A3SS], mutually exclusive exons [MXE], retained intron [RI]), rMATS [50] was used. Briefly, processed RNA-seq reads from nutrient stress experiments were mapped to the genome using STAR [82] and MISO [110] was employed to verify AS events. Iso-Seq reads were also mapped to the genome using STAR [82] to confirm the presence of exons. To evaluate differential exon usage, DEXSeq (version 1.28.3) [111] was used. Exon expression counts for each replicate in nutrient stress experiments were quantified using the Amphidinium genome annotation and BAM files generated from STAR [81] mapping. Default normalization of libraries was performed, and *p* values were corrected using FDR with a *p*-adjust cutoff of < 0.05. Gene ontology term functional enrichment of all genes showing alternative splicing was performed using the GOstats R package [112] and visualized using REVIGO [113]. Figure 4 depicts the results of these analyses.

### PKS protein immunolocalization

Cells grown in normal ASW were first fixed in 2% paraformaldehyde in seawater, washed three times with PBS, and incubated in 50% methanol:PBS (5 min). Cells were then deposited on poly-l-lysine-coated coverslips, blocked with 5% normal goat serum for 1 h, and incubated with primary anti-PKS antibodies (KS and KR) at 1:100 dilution overnight at 4 °C. Cells were then incubated with Alexa Fluor-488-conjugated secondary antibodies for 1 h at room temperature. Coverslips were then mounted with Vectashield on glass slides and observed under a Zeiss Axio-Observer Z1 LSM 780 microscope. Data were collected using ZEN software (version 14.0.8.201). For negative controls, cells were treated with PBS instead of primary antibodies. Stacks were analyzed using ImageJ [114].

## Supplementary information

**Additional file 1: Fig. S1.** Genome and transcriptome features of *A. gibbosum*. **Fig. S2.** Phylogenetic analysis of ketosynthase [KS], acyltransferase [AT] and condensation domains [C] using Bayesian inference. **Fig. S3.** Phylogenetic organization of adenylation domains from dinoflagellates. **Fig. S4.** Global expression profiles and enrichment of differentially expressed genes under nitrogen starvation (q-value < 0.001 and |log2(FC)| > 1). **Fig. S5.** Global expression profile and enrichment of differentially expressed genes under phosphate starvation (q-value < 0.001 and |log2(FC)| > 2). **Fig. S6.** Alignment of functional domains of the *A. gibbosum* homolog. **Fig. S7.** Length, distribution, and enrichment analysis of microRNAs detected from *A. gibbosum*. **Fig. S8.** Mapping of Illumina and Isoseq reads to g70808 and the presence of exons. **Fig. S9.** Immunofluorescent staining of *Amphidinium* with anti-KS and anti-KR antibodies. **Fig. S10.** Genome and transcriptome assembly workflows for *Amphidinium gibbosum*.

**Additional file 2: Supplementary Table 1.** (a) Details of genome assembly based on statistics of scaffolds (b). Annotation statistics for gene models. **Supplementary Table 2.** The 30 most abundant domains in *Amphidinium gibbosum.* **Supplementary Table 3.** *Amphidinium gibbosum* repeat content. **Supplementary Table 4.** Comparison of major repeat content in Symbiodiniaceae and *A. gibbosum.* **Supplementary Table 5.** Top 10 KEGG pathways in *A. gibbosum* transcriptome. **Supplementary Table 6.** Significantly enriched KEGG pathways upregulated or downregulated under N and P starvation. **Supplementary Table 7.** miRNA KEGG pathway target enrichment under nitrogen and phosphate starvation. **Supplementary Table 8.** Details of miRNAs predicted from the *A. gibbosum* genome. **Supplementary Table 9.** Main differentially expressed genes during nutrient starvation in *A. gibbosum*, as shown in Fig. 3a. **Supplementary Table 10.** Annotation of *PKS* and *NRPS* genes under nitrogen and phosphate starvation, as shown in Fig. 3b.

**Additional file 3.** Sequence alignment of the A-domain.

## Data Availability

Sequence data from this study are available in the NCBI Short Read Archive (SRA) Bioproject ID PRJNA551917 [115]. Assembled genome, transcriptome, predicted gene models, and proteins are available at: https://marinegenomics.oist.jp/amphidinium/viewer/download?project_id=83 [116].
