# Supplementary material for: Integrated omics unveil the secondary metabolic landscape of a basal dinoflagellate
Source: BMC Biol. 2020 Oct 13;18:139. doi: 10.1186/s12915-020-00873-6 (PMC7557087; doi:10.1186/s12915-020-00873-6)
Supplement: Supplementary file 1 — Additional file 1: Fig. S1. Genome and transcriptome features of A. gibbosum. Fig. S2. Phylogenetic analysis of ketosynthase [KS], acyltransferase [AT] and condensation domains [C] using Bayesian inference. Fig. S3. Phylogenetic organization of adenylation domains from dinoflagellates. Fig. S4. Global expression profiles and enrichment of differentially expressed genes under nitrogen starvation (q-value < 0.001 and |log2(FC)| > 1). Fig. S5. Global expression profile and enrichment of differentially expressed genes under phosphate starvation (q-value < 0.001 and |log2(FC)| > 2). Fig. S6. Alignment of functional domains of the A. gibbosum homolog. Fig. S7. Length, distribution, and enrichment analysis of microRNAs detected from A. gibbosum. Fig. S8. Mapping of Illumina and Isoseq reads to g70808 and the presence of exons. Fig. S9. Immunofluorescent staining of Amphidinium with anti-KS and anti-KR antibodies. Fig. S10. Genome and transcriptome assembly workflows for Amphidinium gibbosum. [file 12915_2020_873_MOESM1_ESM.pdf]

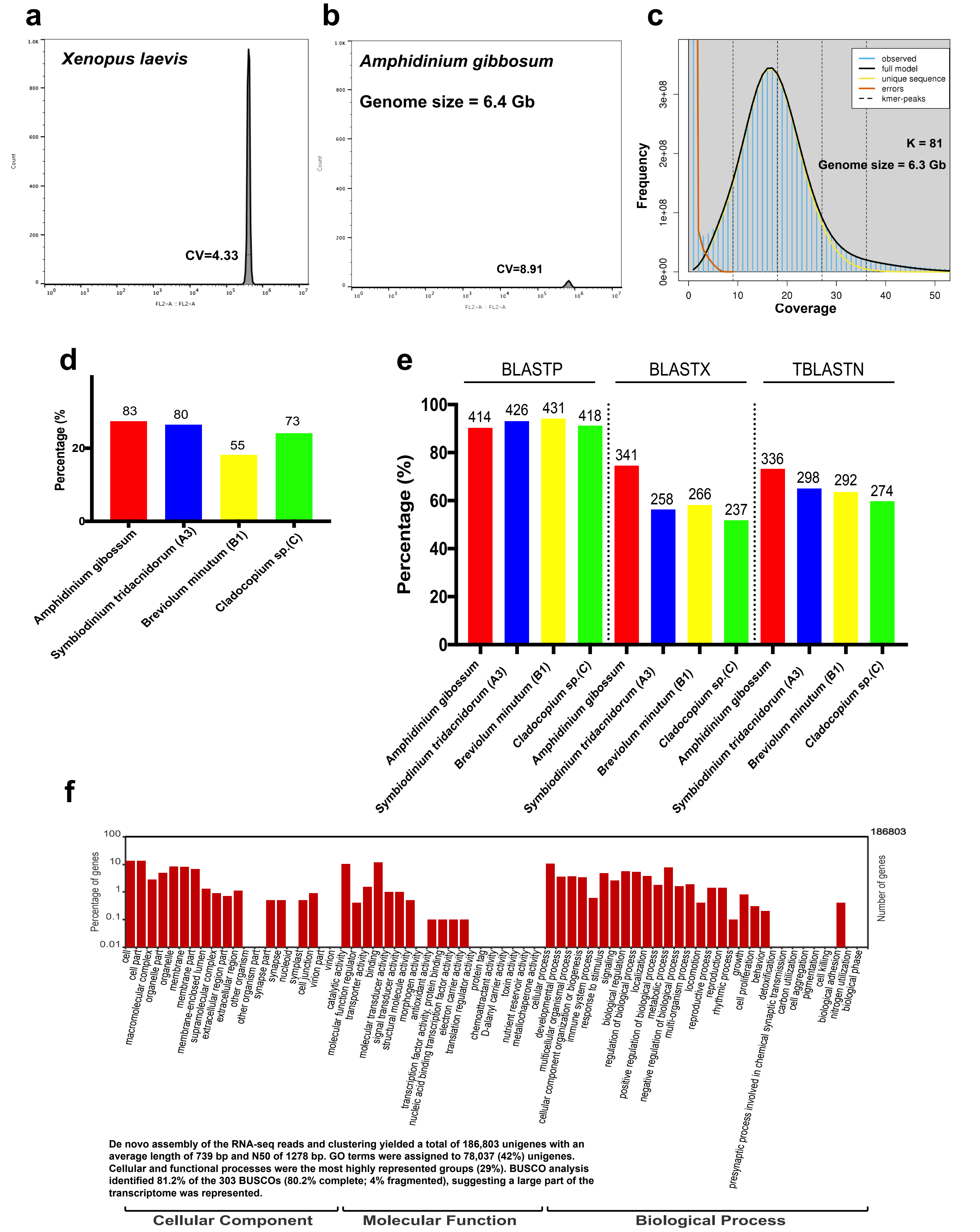

Fig. S1. Genome and transcriptome features of *A. gibbosum*. (a) FACS analysis of 3.1 Gb *Xenopus laevis* genome, (b) *A. gibossum* haploid genome is approximately 6.4 Gb in size, which matches (c) Genomescope estimation at k= 81. CV is the Coefficient of Variation. (d) Recovery of 303 BUSCO genes in *Amphidinium gibbosum*, and (e) recovery of 458 CEGMA genes based on several BLAST analyses using BLASTP for predicted proteins and genome scaffolds for BLASTX and TBLASTN, respectively. (f) Gene annotation of *A.gibbosum* unigenes using gene ontology (GO).

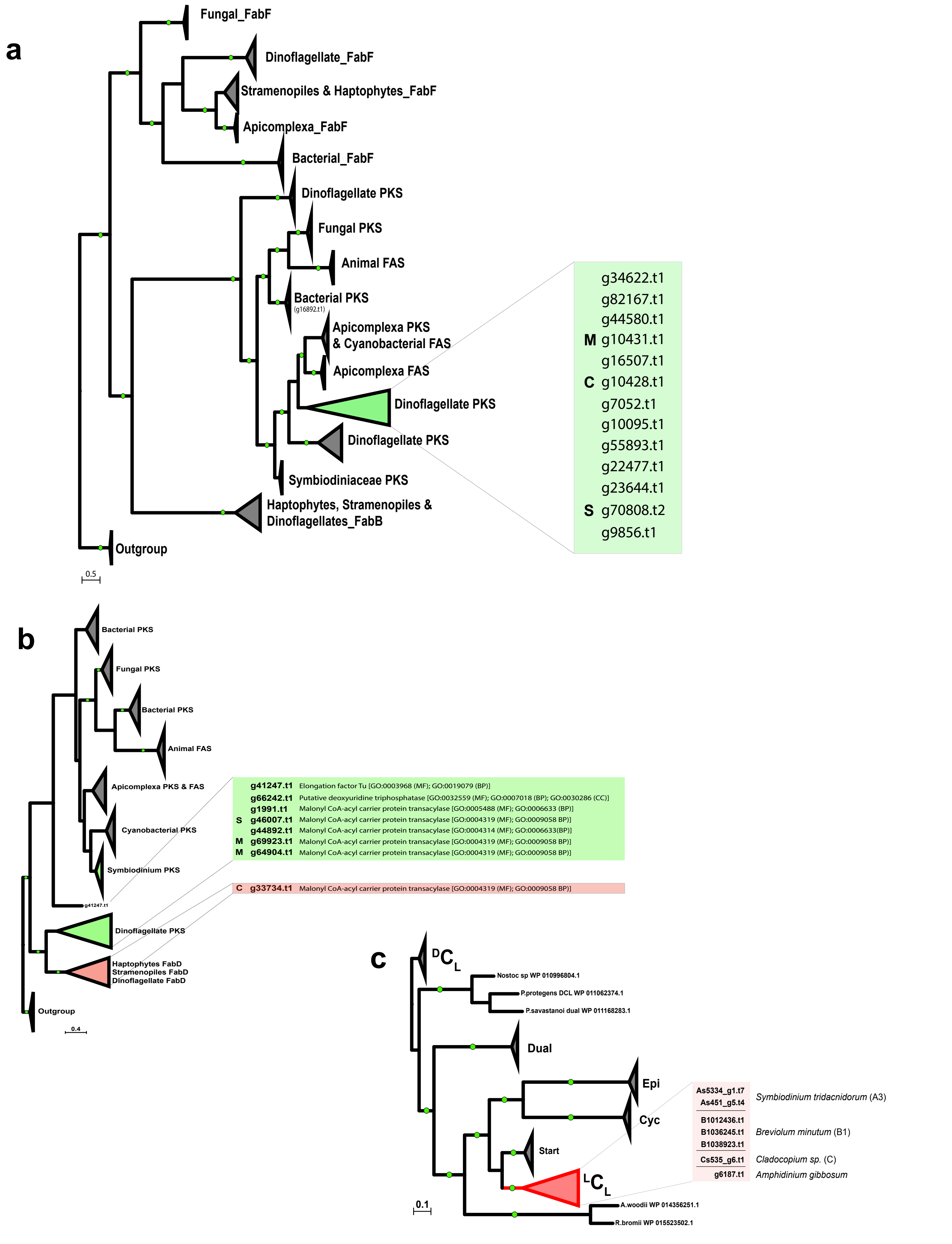

Fig. S2. Phylogenetic analysis of (a) ketosynthase [KS], (b) acyltransferase [AT] and (c) condensation domains [C] using Bayesian inference. Green circles indicate a probability ≥ 0.75.
C, M and S depicts chloroplast, mitochondria, and secretory signal peptide respectively.

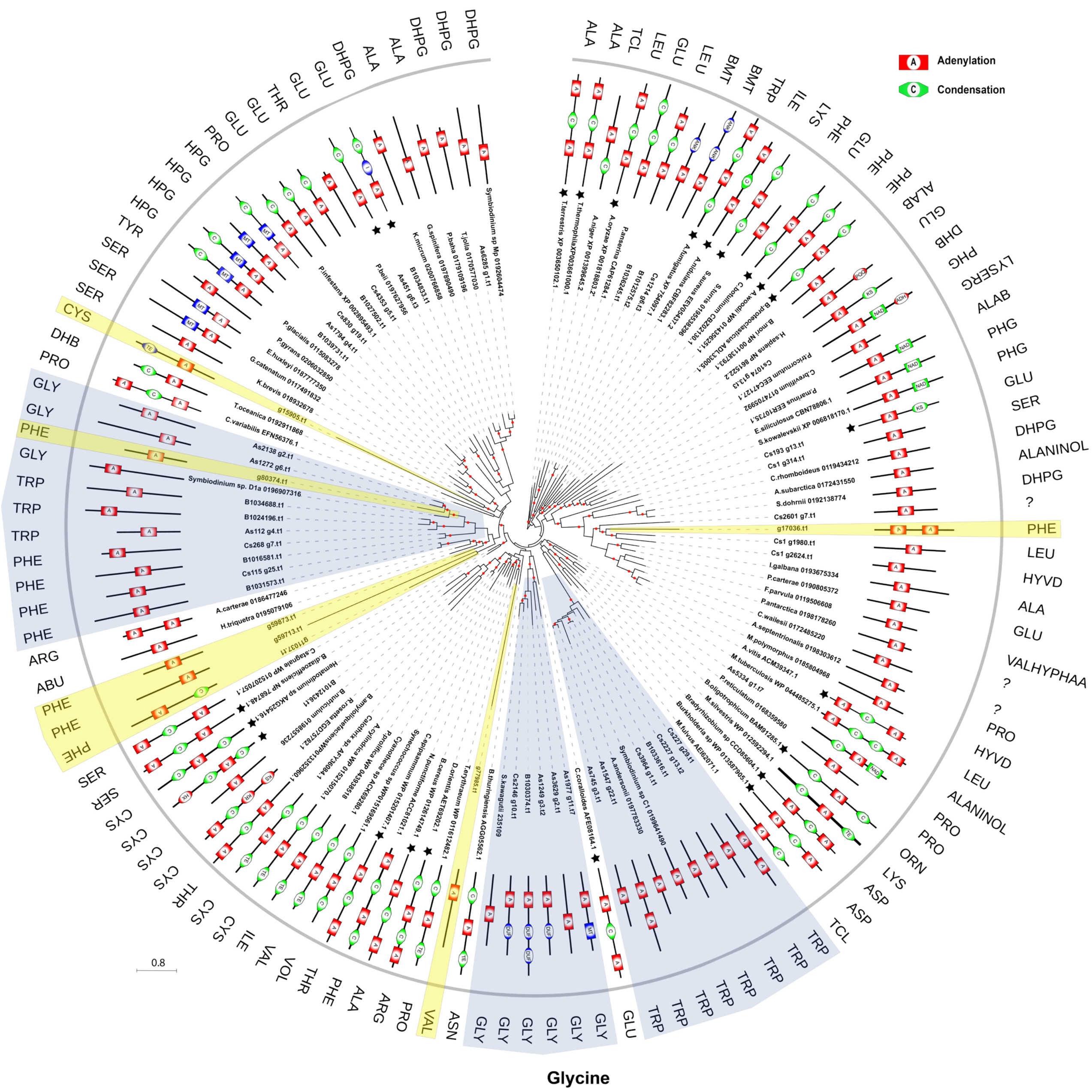

Fig. S3. Phylogenetic organization of adenylation domain from dinoflagellates.
The blue and yellow areas are Symbiodiniaceae and *A. gibbosum* adenylation enzyme sequences, respectively. Red circles indicate a posterior probability ≥ 0.75 using Bayesian inference. Asterisks near sequences indicate partial domain organization.

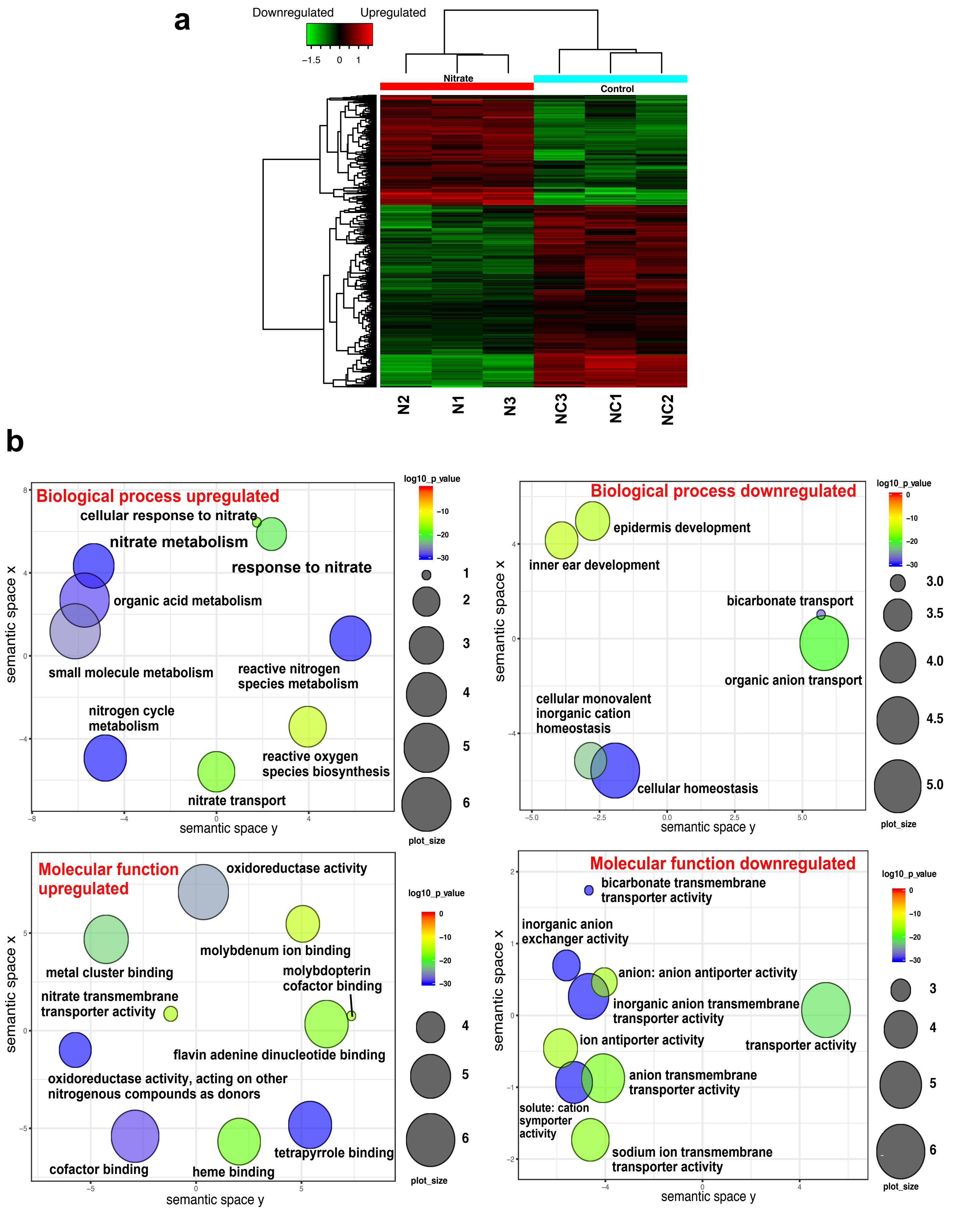

Fig. S4. (a) Global expression profile of differentially expressed genes under nitrogen starvation (q-value < 0.001 and |log2(FC)| > 1). (b) Functional enrichment of GO-terms analyzed with topGO and summarized with REVIGO showing top 10 hits in nitrogen starvation. Scatterplots summarize the GO terms based on semantic similarities. Similar GO terms remain in close proximity together. Bubble color represent p < 0.001 while circle size shows the frequency of the GO term.

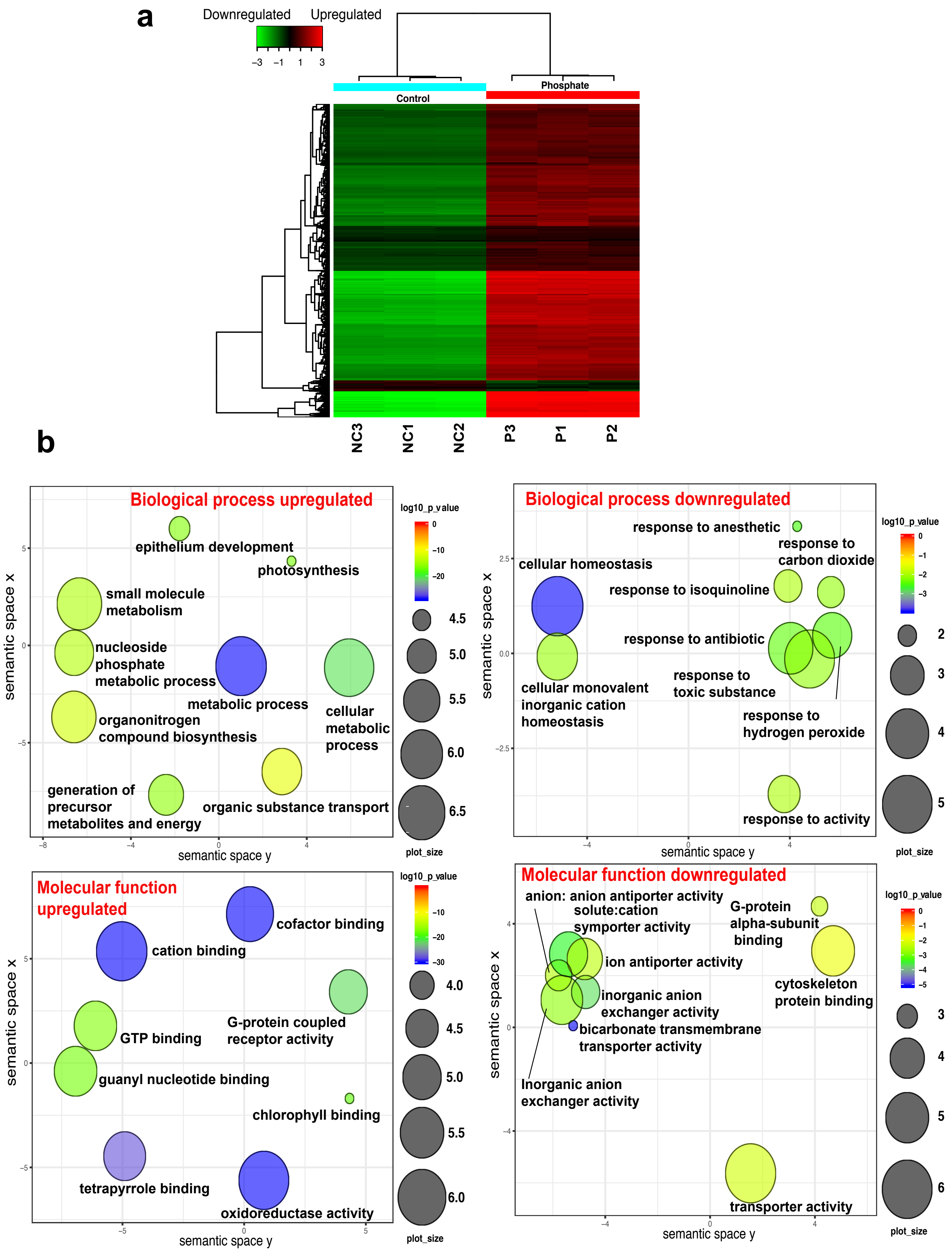

Fig. S5. (a) Global expression profile of differentially expressed genes under phosphate starvation (q-value < 0.001 and |log2(FC)| > 2). (b) Functional enrichment of GO-terms analyzed with topGO and summarized with REVIGO for phosphate starvation showing top 10 hits. Scatterplots summarize the GO terms based on semantic similarities; Similar GO terms remain in close proximity together. Bubble color represents p < 0.001 while circle size shows the frequency of the GO term.

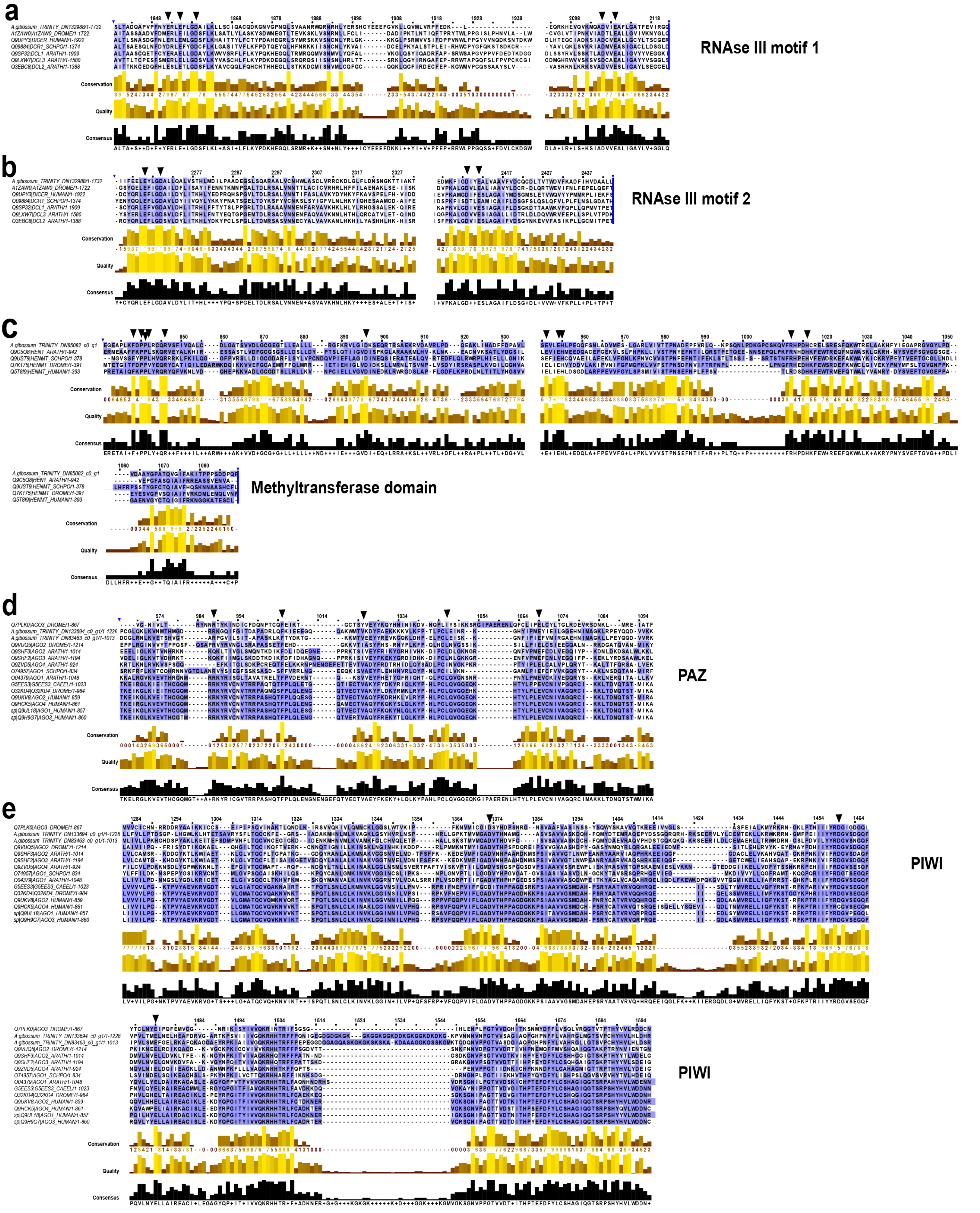

Fig. S6. Alignment of functional domains of the A. gibbosum homolog.
Alignments depicts Dicer RNAse III (a) motif 1 and (b) motif 2, (c) small RNA 2'-O-methyltransferase (HEN1), argonaute protein, (d) PAZ domain and (e) PIWI domain with homologs from model organisms.
Key functional residues are depicted with black triangle.

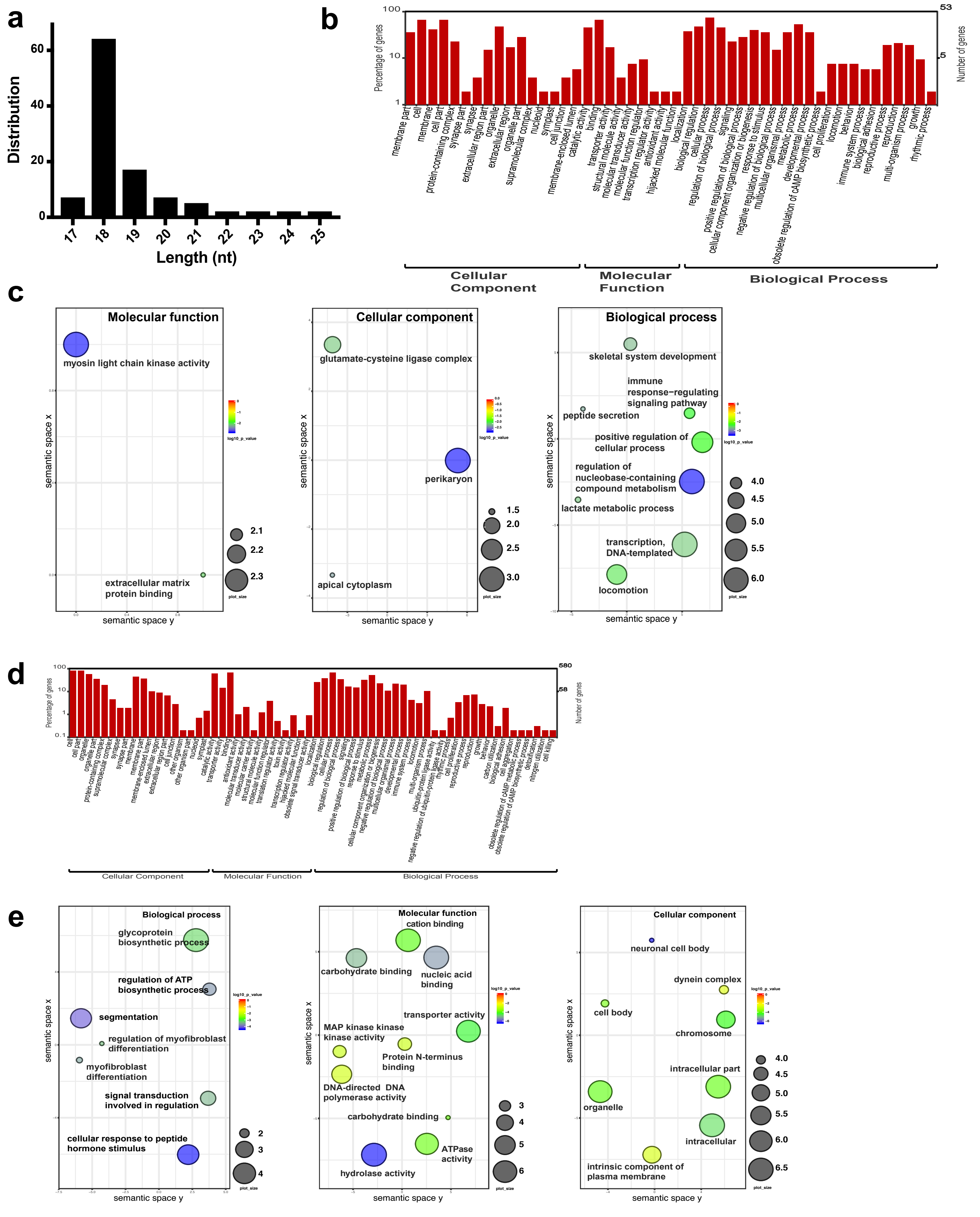

Fig. S7. (a) Length and distribution of microRNAs detected from *A. gibbosum* (b) Gene ontology classification for 303 predicted target unigenes of one differentially expressed miRNA under nitrogen starvation in three GO categories. Only 53 genes had GO annotations. (c) Functional enrichment of GO-terms analyzed with topGO and summarized with REVIGO. Bubble color represent p < 0.01 while circle size shows the frequency of the GO term. (d) Gene ontology classification for 2711 predicted target unigenes of two differentially expressed miRNA under phosphate starvation in three GO categories. Only 580 genes had GO annotations. (e) Functional enrichment of GO-terms analyzed with topGO and summarized with REVIGO. Bubble color represent p < 0.01 while circle size shows the frequency of the GO term.

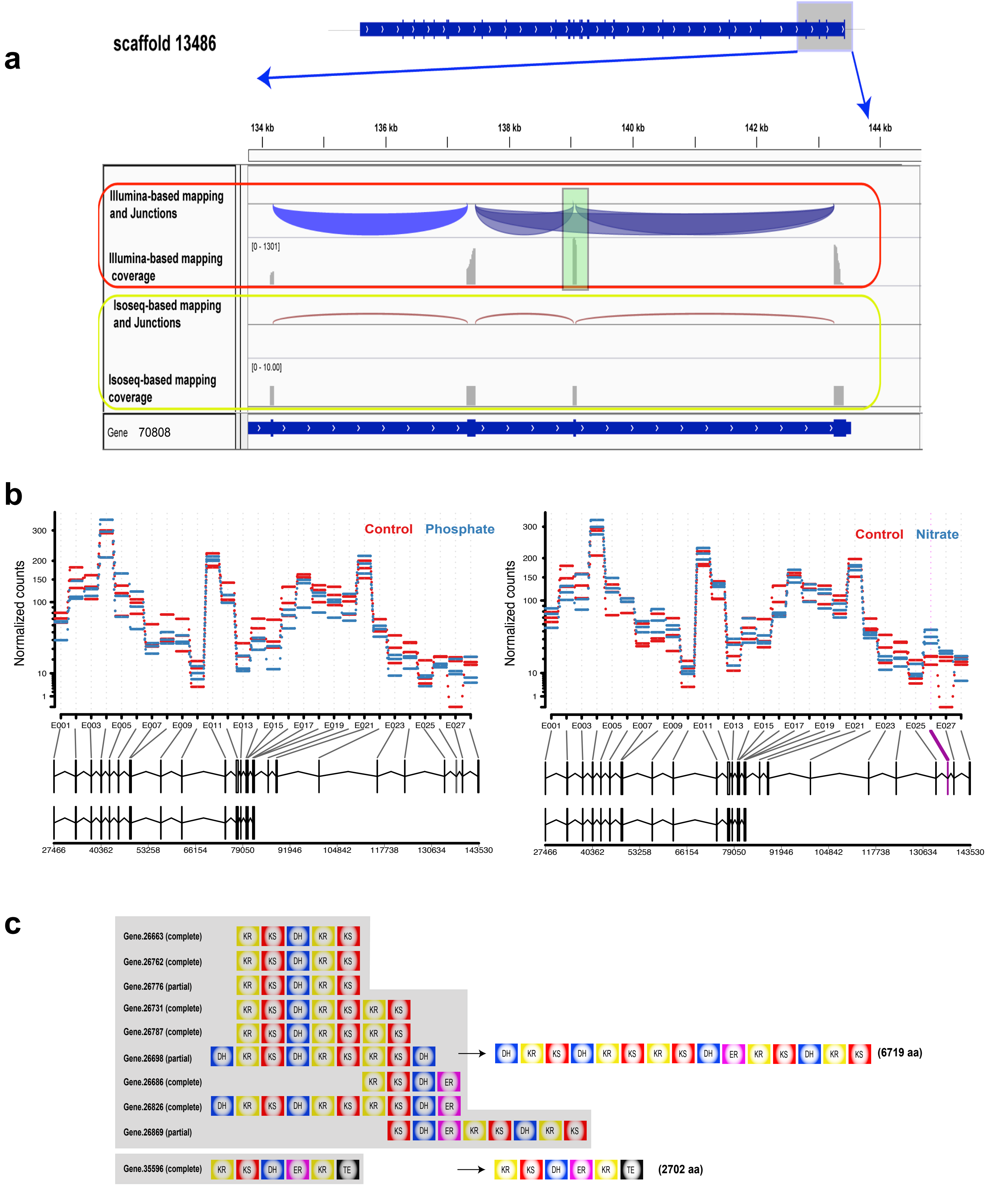

Fig. S8. (a) Plot showing mapping of Illumina and Isoseq reads to g70808 and the presence of exons. Illumina reads mapping are depicted in red box while Isoseq reads mapping are in yellow box. Reads show evidence of skipped exon (shown in green box). Isoseq read coverage is too low to show such skipped exon event. (b). Gene models showing alternative splicing in g70808. Plots show counts after being normalized in each replicate of phosphate and nitrogen starvation experiment. Significant differentially expressed exons are coloured in magenta (p.adjust < 0.05). (c) PKS transcripts recovered from Iso-Seq showing evidence of expression of multifunctional PKS transcripts. Length are expressed in amino acids. (KS, ketosynthase; KR, ketoreductase, DH; dehydratase, ER, enoylreductase; TE, thioesterase).

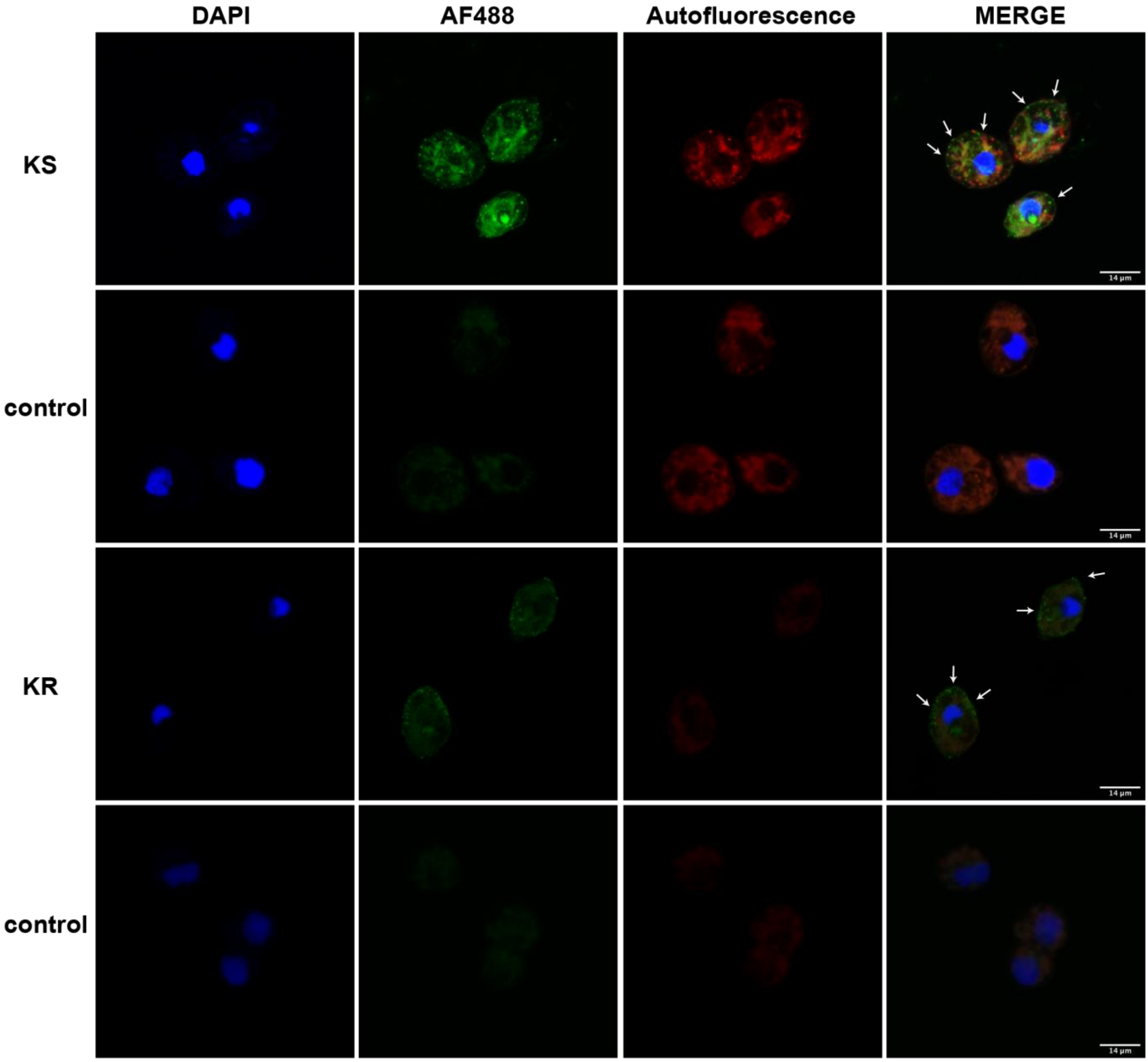

Fig. S9. Immunofluorescent staining of Amphidinium with anti-KS and anti-KR antibodies. Confocal images (63x) of antibodies show localization of KS and KR proteins indicated with white arrows. Nuclei are stained blue with DAPI. KS proteins are in green, and merged images of nuclei and KS and KR protein staining, respectively. Scale bars are 14 µm in the panels.

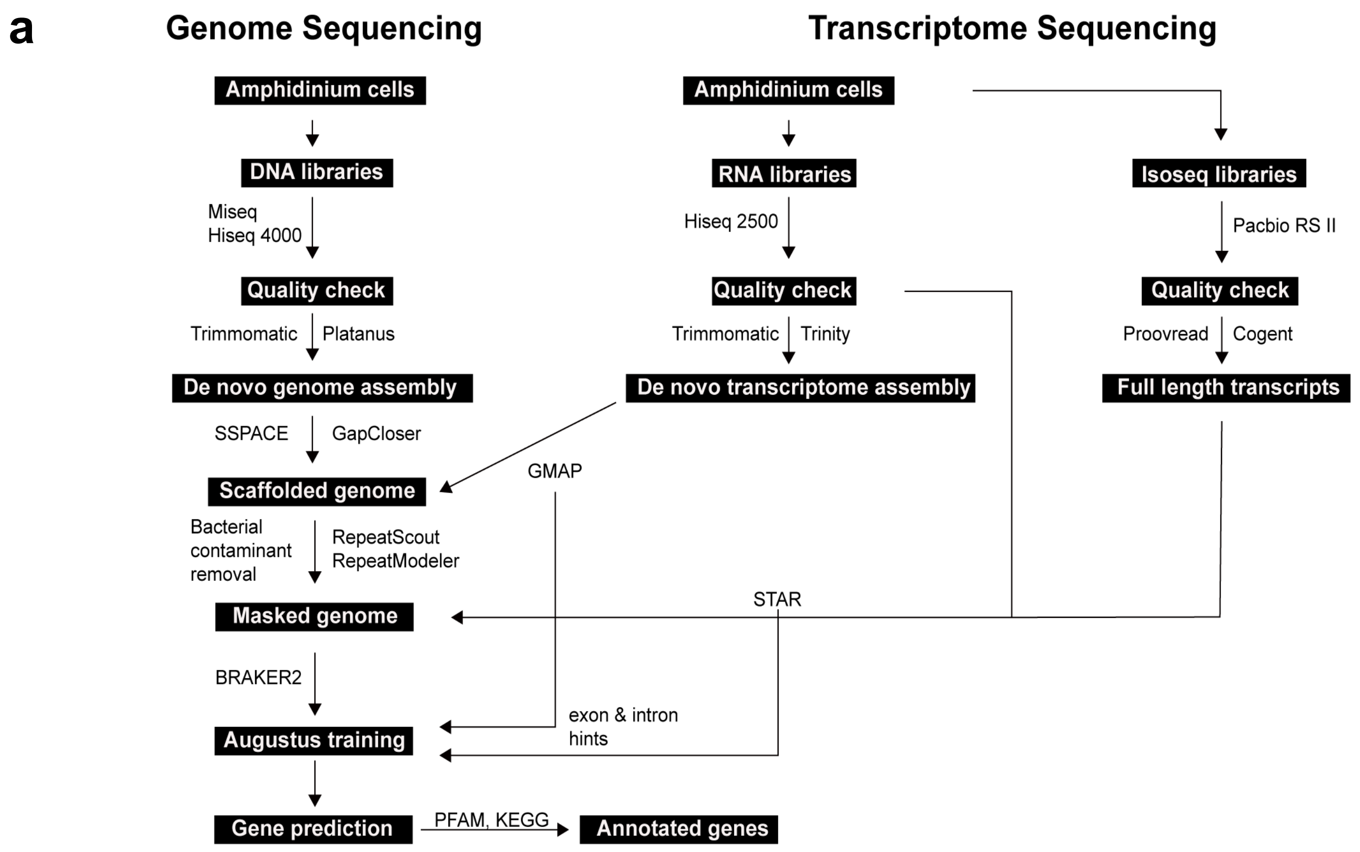

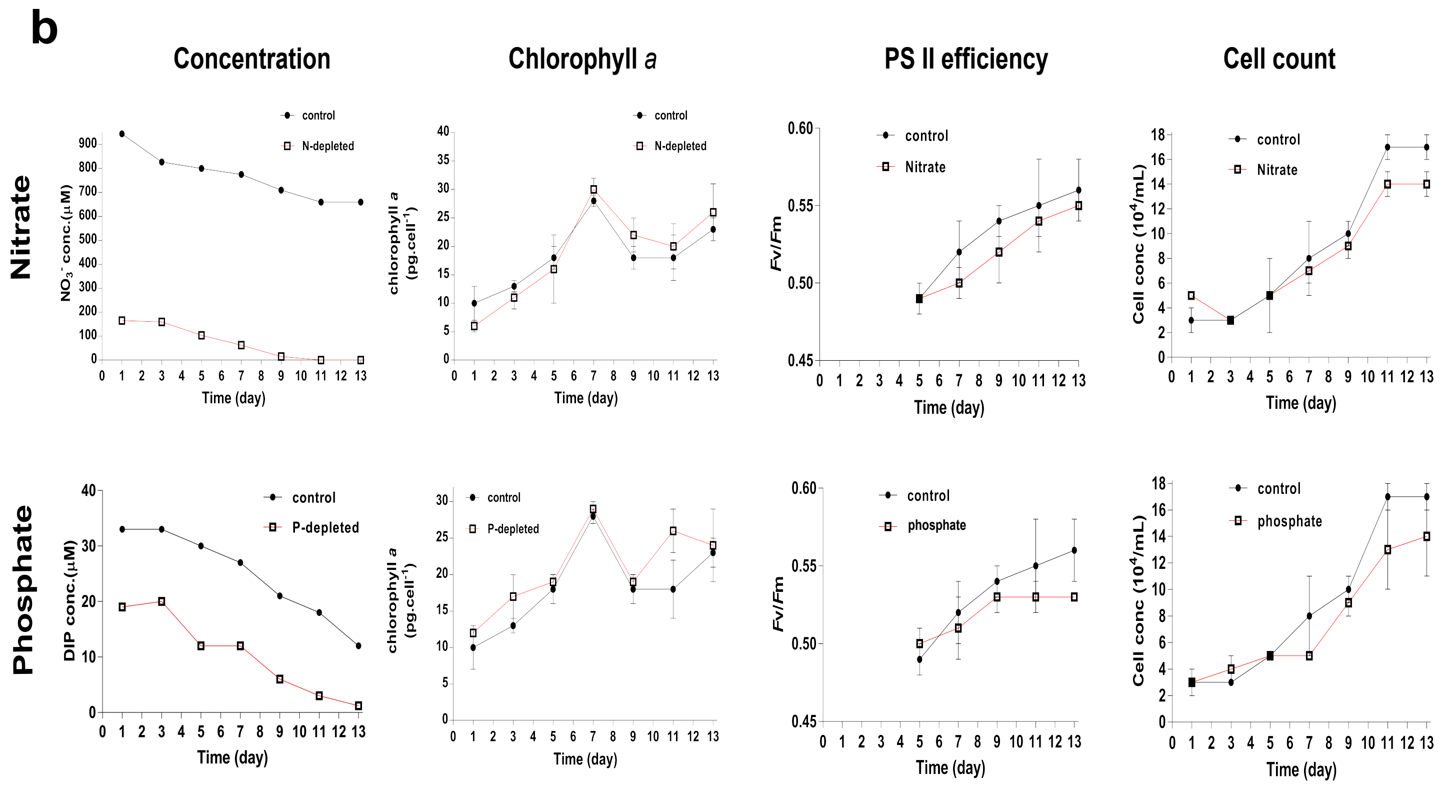

Fig. S10. (a) Genome and transcriptome assemblies' workflow for *Amphidinium gibbosum*. (b) Physiological parameters of Amphidinium gibbosum under nitrate and phosphate starvation conditions. Results are mean ± SE of 3 replicates (except for nitrate/phosphate concentration).
