# Supplementary material for: Integrated omics unveil the secondary metabolic landscape of a basal dinoflagellate
Source: BMC Biol. 2020 Oct 13;18:139. doi: 10.1186/s12915-020-00873-6 (PMC7557087; doi:10.1186/s12915-020-00873-6)
Supplement: Supplementary file 2 — Additional file 2: Supplementary Table 1. (a) Details of genome assembly based on statistics of scaffolds (b). Annotation statistics for gene models. Supplementary Table 2. The 30 most abundant domains in Amphidinium gibbosum. Supplementary Table 3. Amphidinium gibbosum repeat content. Supplementary Table 4. Comparison of major repeat content in Symbiodiniaceae and A. gibbosum. Supplementary Table 5. Top 10 KEGG pathways in A. gibbosum transcriptome. Supplementary Table 6. Significantly enriched KEGG pathways upregulated or downregulated under N and P starvation. Supplementary Table 7. miRNA KEGG pathway target enrichment under nitrogen and phosphate starvation. Supplementary Table 8. Details of miRNAs predicted from the A. gibbosum genome. Supplementary Table 9. Main differentially expressed genes during nutrient starvation in A. gibbosum, as shown in Fig. 3a. Supplementary Table 10. Annotation of PKS and NRPS genes under nitrogen and phosphate starvation, as shown in Fig. 3b. [file 12915_2020_873_MOESM2_ESM.pdf]

Supplementary Table 1. (a) Details of genome assembly based on statistics of scaffolds (b) Annotation statistics for gene models.

a

# contigs (>= 1000 bp)	174,692
# contigs (>= 5000 bp)	108,913
# contigs (>= 10000 bp)	76,033
# contigs (>= 25000 bp)	46,743
# contigs (>= 50000 bp)	34,084
Total length (>= 0 bp)	7,564,364,748
Total length (>= 1000 bp)	6,949,758,089
Total length (>= 5000 bp)	6,825,375,768
Total length (>= 10000 bp)	6,574,923,633
Total length (>= 25000 bp)	6,102,117,029
# contigs	298,063
Total length (>= 50000 bp)	5,649,811,202
Largest contig	3,442,467
Total length	7,034,147,423
GC (%)	47.09
N50	166,499
N75	71,058
L50	12,063
L75	27,848

b

	Number	Percentage
Total number of gene models	85,139	100
Number of gene models with Swiss-Prot top hits	18,015	21.1
Number of gene models with TrEMBL top hits	41,117	48.3
Number of gene models with association with KEGG orthologs	3,827	4.5
Number of gene models with annotated Pfam domains	5,442	6.4

Supplementary Table 2. Top 30 abundant domains in *Amphidinium gibbosum*

Pfam domain	Pfam ID	Function	Agb	Str	Bmi	Cla	Fka	Pfa	Ehu	Tth	Tbr	Gth
DUF4116	PF13475.5	Domain of unknown function (DUF4116)	13747	856	525	672	233	0	17	0	0	66
LRR_8	PF13855.5	Leucine rich repeat	5987	664	1963	2342	677	40	1003	296	408	1077
LRR_4	PF12799.6	Leucine Rich repeats (2 copies)	3437	1062	1427	2720	930	98	802	1702	686	1546
Ank_3	PF13606.5	Ankyrin repeat	2063	10804	4185	7439	2496	68	1444	381	153	1717
Ank	PF00023.29	Ankyrin repeat	2023	0	0	7044	2383	52	1341	322	132	1580
Ank_5	PF13857.5	Ankyrin repeats (many copies)	1951	9823	3716	6663	2123	60	1323	302	110	1464
Ank_4	PF13637.5	Ankyrin repeats (many copies)	1851	8877	3605	6115	2095	52	1246	352	112	1444
RVT_1	PF00078.26	Reverse transcriptase (RNA-dependent DNA polymerase)	1741	1143	369	1016	472	0	7	7	1	52
TPR_1	PF00515.27	Tetratricopeptide repeat	1459	1399	1224	2351	933	0	731	2724	0	1405
Ank_2	PF12796.6	Ankyrin repeats (3 copies)	1451	6962	2802	4663	1567	45	974	253	86	1041
PPR	PF01535.19	PPR repeat	1311	10508	5093	6033	820	8	417	104	84	198
TPR_2	PF07719.16	Tetratricopeptide repeat	1262	1752	1428	2485	1050	112	915	2943	329	1524
PPR_2	PF13041.5	PPR	1311	10508	5113	6025	774	16	373	22	93	269
TPR_11	PF13414.5	TPR repeat	1150	531	414	530	356	17	285	1609	152	856
Ephrin_rec_like	PF07699.12	Putative ephrin-receptor like	1092	1069	753	1154	355	53	69	360	0	1441
EF-hand_1	PF00036.31	EF hand	1089	3441	2726	2744	929	102	647	798	126	847
TPR_17	PF13431.5	Tetratricopeptide repeat	1078	488	451	1029	503	206	413	1890	110	831
EF-hand_6	PF13405.5	EF-hand domain	1057	3454	2718	2783	942	105	645	823	138	809
TPR_14	PF13428.5	Tetratricopeptide repeat	1039	843	876	1789	639	31	723	1666	264	1128
Pkinase	PF00069.24	Protein kinase domain	993	1475	989	1088	510	206	740	1894	232	603
TPR_8	PF13181.5	Tetratricopeptide repeat	971	977	735	1823	761	54	548	2683	194	1193
LRR_1	PF00560.32	Leucine Rich Repeat	933	360	573	2448	670	0	655	37	49	817
PPR_1	PF12854.6	PPR repeat	884	6095	3077	3616	470	8	334	17	43	196
PPR_3	PF13812.5	Pentatricopeptide repeat domain	830	6564	3445	4014	490	5	241	17	64	210
WD40	PF00400.31	WD domain, G-beta repeat	770	1728	980	1450	539	325	980	1793	573	1865
Pkinase_Tyr	PF07714.16	Protein tyrosine kinase	753	1287	852	915	424	105	657	1353	228	575
LRR_6	PF13516.5	Leucine Rich repeat	716	1539	1149	1680	776	22	1761	10655	290	1057
EF-hand_7	PF13499.5	EF-hand domain pair	710	2413	1889	1851	593	72	397	581	105	574
RCC1_2	PF13540.5	Regulator of chromosome condensation (RCC1) repeat	705	8647	2221	6193	496	57	406	284	57	519
TPR_12	PF13424.5	Tetratricopeptide repeat	680	1482	1196	2448	855	33	662	2084	174	1005

Agb, *Amphidinium gibossum*; Str, *Symbiodinium tridacnidorum* (A3); Bmi, Breviolum minutum(B1); Cla, *Cladocopium* sp. (C); Fka, *Fugacium kawagutii* (F); Pfa, *Plasmodium falciparum*; Ehu, *Emiliania huxleyi*; Tth, *Tetrahymena thermophila*; Tbr, *Trypanosoma brucei*; Gth, *Guillardia theta*

Supplementary Table 3. *Amphidinium gibbosum* repeat content.

Category	Element	Number of occurrences	Number of bp covered
DNA transposon	Academ-1	121	4703
	Academ-2	3	127
	Academ-H	2	83
	CMC-Chapaev	1050	43084
	CMC-Chapaev-3	115	4641
	CMC-EnSpm	19149	1767122
	CMC-Mirage	1	40
	CMC-Transib	2682	144587
	Crypton	361	13437
	Crypton-A	73	2792
	Crypton-C	2	124
	Crypton-F	13	606
	Crypton-H	542	24172
	Crypton-S	20	1144
	Crypton-V	1070	37362
	Crypton-X	2	115
	Dada	707	29827
	Ginger	3188	249028
	IS3EU	596	22225
	Kolobok	27	1104
	Kolobok-E	7	403
	Kolobok-Hydra	1561	101722
	Kolobok-T2	1905	99366
	MULE-F	7	389
	MULE-MuDR	19897	1437967
	MULE-NOF	85	3786
	Maverick	2912	157819
	Merlin	673	24815
	Novosib	1409	152653
	P	1882	82424
	P-Fungi	6	207
	PIF-HarbS	8	307
	PIF-Harbinger	3838	174415
	PIF-ISL2EU	157	6661
	PIF-Spy	560	27903
	PiggyBac	211	9005
	PiggyBac-A	1	42
	PiggyBac-X	44	3446
	Sola-1	951	67602
	Sola-2	147	6910
	Sola-3	630	69590
	TcMar	2675	146449
	TcMar-Ant1	8	391
	TcMar-Cweed	3	111
	TcMar-Fot1	1052	62995
	TcMar-Gizmo	14	2065

Supplementary Table 3. Continued

	TcMar-ISRm11	242	8716
	TcMar-Mariner	293	15801
	TcMar-Pogo	15	738
	TcMar-Sagan	3	127
	TcMar-Stowaway	105	4630
	TcMar-Tc1	3364	211876
	TcMar-Tc2	49	4872
	TcMar-Tc4	33	1252
	TcMar-Tigger	18	776
	TcMar-m44	8	255
	Zator	8	378
	Zisupton	4866	303673
	hAT	1004	49962
	hAT-Ac	5088	329748
	hAT-Blackjack	291	12307
	hAT-Charlie	2465	157368
	hAT-Pegasus	95	6023
	hAT-Restless	3	87
	hAT-Tag1	910	40818
	hAT-Tip100	1582	63810
	hAT-hAT1	12	763
	hAT-hAT19	13	454
	hAT-hAT5	16	570
	hAT-hAT6	2	61
	hAT-hATm	340	12001
	hAT-hATw	74	3221
	hAT-hATx	3	120
	hAT-hobo	11	648
**LINE**	CR1	352	51472
	CR1-Zenon	20	774
	CRE	107	5717
	CRE-Ambal	53	3285
	CRE-Odin	3	201
	Deceiver	1	17
	Dong-R4	17	566
	Dualen	7	380
	Genie	2	177
	I	138	6550
	I-Jockey	2077	164909
	L1	3393	225473
	L1-DRE	24	1311
	L1-Tx1	1913	112143
	L1-Zorro	3	51
	L2	4005	269015
	Penelope	3456	187633
	Proto1	17	1088
	Proto2	9	436

Supplementary Table 3. Continued

	R1	1492	134471
	R1-LOA	16	706
	R2	168	11286
	R2-Hero	66	3347
	R2-NeSL	332	36457
	RTE-BovB	188	8569
	RTE-RTE	21	553
	RTE-X	195	12313
	Rex-Babar	94	4096
	Tad1	52	2487
**LTR**	Bhikhari	11	473
	Caulimovirus	35	1365
	Copia	3691	179769
	DIRS	1735	79064
	ERV-Foamy	3	86
	ERV-Lenti	1	69
	ERV1	3823	201937
	ERV4	67	2668
	ERVK	1564	78030
	ERVL	169	7493
	ERVL-MaLR	7	418
	Gypsy	16541	1240334
	Ngaro	483	21957
	Pao	762	36725
	Viper	5	234
**Other**	DNA_virus	9	393
**RC**	Helitron	4753	275930
	Helitron-2	60	2844
**Retroposon**	SVA	9	604
**SINE**	5S	1	64
	5S-Deu-L2	1	43
	5S-RTE	5	146
	7SL	2	62
	B2	1	28
	B4	55	2381
	ID	23	730
	MIR	3	73
	RTE	2	96
	RTE-BovB	2	130
	U	1	47
	tRNA	740	39558
	tRNA-5S	1	19
	tRNA-7SL	5	194

Supplementary Table 3. Continued

	tRNA-CR1	2	49
	tRNA-Ceph-RTE	4	260
	tRNA-Core	97	3206
	tRNA-Core-RTE	3	170
	tRNA-Deu	5	207
	tRNA-Deu-L2	2	138
	tRNA-I	6	201
	tRNA-L1	2	52
	tRNA-L2	7	272
	tRNA-Mermaid	2	151
	tRNA-Meta	20	724
	tRNA-RTE	5	269
	tRNA-Sauria	1	41
	tRNA-V-CR1	2	41
	TATE	1	55
	Y-chromosome	1	64
	centromeric	1	13
**Unspecified**		**9789040**	**2068177725**
**Total interspersed**		**9942170**	**2078587436**
**Low_complexity**		**331120**	**29212862**
**RNA**		**1**	**85**
**Satellite**	5S	43	2694
	acro	39	3443
	macro	27	786
	telo	3	53
**Simple_repeat**		**2230179**	**148606652**
**rRNA**		**469**	**24880**
**snRNA**		**19**	**566**
**tRNA**		**221**	**6139**
**Total**		**12514653**	**2257532404**

Supplementary Table 4. Comparison of major repeat content in Symbiodiniaceae and *A. gibbosum*

	Symbiodinium microadriaticum[a]		*Breviolum minutum*[b]		*Amphidinium gibossum*[c]	
	Number of occurrences	Percentage of genome	Number of occurrences	Percentage of genome	Number of occurrences	Percentage of genome
**LTR Retrotransposon**						
Copia	6723	0.003	13550	0.3	3691	0.002
Gypsy	11722	0.012	12970	0.272	16541	0.019
**LINE**						
R2	73	0.003	7071	0.133	168	0.001
Jockey	2960	0.012	5001	0.105	2077	0.002
R1	1043	0.004	3027	0.055	1492	0.002
L1	175702	0.769	2279	0.046	3393	0.003
CR1	22074	0.123	1135	0.027	352	0.001
**DNA transposons**						
MuDR	7580	0.032	16701	0.35	19897	0.022
Maverick	NR	NR	1114	0.035	2912	0.002
Novosib	5022	0.017	1100	0.02	1409	0.002
EnSpm	80349	0.247	1095	0.021	19149	0.027
hAT	15642	0.072	824	0.015	1004	0.001

[a] [9]
[b] [4]
[c] (This study)

Supplementary Table 5. Top 10 KEGG pathways in *A. gibbosum* transcriptome. Numbers of proteins recovered are indicated in parenthesis. KEGG pathway classification recovered 422 pathways, of which metabolic and biosynthesis of secondary metabolites pathways accounted for 1187 proteins.

**Top 10 represented KEGG pathways**
ko01100 Metabolic pathways (841)
ko01110 Biosynthesis of secondary metabolites (346)
ko01130 Biosynthesis of antibiotics (233)
ko01120 Microbial metabolism in diverse environments (193)
ko01230 Biosynthesis of amino acids (109)
ko03010 Ribosome (105)
ko03040 Spliceosome (94)
ko01200 Carbon metabolism (91)
ko00230 Purine metabolism (77)
ko04141 Protein processing in endoplasmic reticulum (75)

Supplementary Table 6. (a) Significantly enriched KEGG pathways upregulated under N starvation**,** (b) downregulated under N starvation**,** and (c) upregulated under P starvation.

a

Pathway	Gene count	Fold enrichment	*p*-value (Fisher's Exact Test)
Nitrogen metabolism	6	234	1.70E-05

b

Pathway	Gene count	Fold enrichment	*p*-value (Fisher's Exact Test)
Bile secretion	5	118.3	1.10E-06
Proteoglycans in cancer	2	49.3	6.70E-04
Pancreatic secretion	4	78.9	2.40E-04

c

Pathway	Gene count	Fold enrichment	*p*-value (Fisher's Exact Test)
Ribosome	25	2.5	3.70E-07
Metabolic pathways	78	1.5	6.80E-06
Biosynthesis of secondary mebolites	38	1.5	2.00E-03
Inositol phosphate metabolism	10	2.4	1.70E-03
Purine metabolism	13	2.1	3.00E-03
mRNA surveillance pathway	12	2.0	6.40E-03
Carbon metabolism	12	2.0	6.40E-03
Pyrimidine metabolism	10	2.3	3.40E-03
Glyoxylate and dicarboxylate metabolism	6	3.1	2.30E-03
Biosynthesis of antibiotics	11	2.1	5.00E-03
Endocytosis	16	1.7	1.10E-03
Insulin signaling pathways	7	2.6	6.10E-03
Proteoglycans in cancer	6	2.7	6.90E-03
Glutamatergic synapse	8	2.2	1.00E-02
Neuroactive ligand-receptor interaction	8	2.2	1.00E-02
Biosynthesis of amino acids	12	1.7	3.10E-02
Ascorbate and aldarate metabolism	5	2.6	1.90E-02
Ubiquitin mediated proteolysis	5	2.6	1.90E-02
Phosphatidylinositol	8	1.9	3.00E-02

Supplementary Table 7. (a) miRNA KEGG pathway target enrichment under nitrogen starvation, and **(**b) miRNA KEGG pathway target enrichment under phosphate starvation.

a

Term	Fold Enrichment	Fisher's Exact Test
Pyruvate metabolism	38.4	0.001
Dilated cardiomyopathy	48	0.00063
GABAergic synapse	24	0.0028
Hypertrophic cardiomyopathy (HCM)	64.1	0.00031

b

Term	Fold Enrichment	Fisher's Exact Test
Fructose and mannose metabolism	7.7	0.0047
Proteoglycans in cancer	5.8	0.012
N-Glycan biosynthesis	5.8	0.012
Hungtington's disease	4.7	0.0079

Supplementary Table 8. Details of miRNAs predicted from the *A. gibbosum* genome.

Provisional id	miRBase miRNA with the same seed	Consensus mature sequence	Consensus star sequence	Precursor coordinate
agi-miR-12395-3p	mdo-miR-12395-3p	cggcccagaacagccccca	ggggguguuuugggccuuu	scaffold19923|size108223:73026..73085:-
agi-miR-6994-5p	mmu-miR-6994-5p	gcaaaggugcugccagca	cugccagcacccuugcca	scaffold2148|size380143:156204..156250:+
agi-miR-1741	gga-miR-1741	uuggcucugaggguuggguuuaggg	ccuugaccacucgggcagacu	scaffold191180|size872:669..734:-
agi-miR-6760-3p	hsa-miR-6760-3p	acacugucgugacugcau	gcaaucgcuacacuguag	scaffold1287|size448889:341824..341891:+
agi-miR-4128-3p	cin-miR-4128-3p	cuugaggugcugccauca	augcagcgcacucggagga	scaffold27849|size71045:16029..16079:+
agi-miR1917	sly-miR1917	guuaauaacuuugagcau	agucaaaguuauuaacug	scaffold7288|size226509:34450..34524:+
agi-miR-7286-5p	mdo-miR-7286-5p	gggagcuugguugggcuu	gcuugguugggcuuggu	scaffold12408|size163128:29551..29630:+
agi-miR-1936	mmu-miR-1936	caacugacucuaaucugac	gggguucagagucaauuuug	scaffold9133|size199635:133828..133874:-
agi-miR-11947a-3p	pte-miR-11947a-3p	guaacagaacagccccca	gggcuuuccaauuacug	scaffold1764|size407553:86669..86743:-
agi-miR-10299	tcf-miR-10299	gucggcaccgaaaugcuugcuu	aaaugccuuauggucguggacgu	scaffold6306|size244690:110424..110490:-
agi-miR-9622-5p	cli-miR-9622-5p	gggggguuaaaaagcucg	agcaaaaaugagccccucc	scaffold68239|size13100:8797..8867:-
agi-miR408-5p	ptc-miR408-5p	uggggaacuaugccugaga	uuauuuggauuucccaug	scaffold3262|size327365:299650..299713:+
agi-miR-3823-5p	tca-miR-3823-5p	caaggucgacaugcauagc	cuugucuguugaucuucuu	scaffold2982|size339141:135698..135737:+
agi-miR2633	mtr-miR2633	cgacauuucaucuccgugag	cgcggaauuugucacu	scaffold6415|size242572:183420..183479:-
agi-miR11500	pab-miR11500	gaugccuguugugucugu	agaucaacaggagagua	scaffold104930|size6504:1355..1411:-
agi-miR-B6-3p	bhv1-miR-B6-3p	agucuccgggacaaacaggaaccg	ggacccgucccugggacaaa	scaffold4733|size281424:46153..46235:+
agi-miR-3009	hma-miR-3009	cuucaucaccuccuuucu	aaaggaggugauuuuuca	scaffold1110|size467226:132282..132355:-
agi-miR-5918d	hco-miR-5918d	gucggcuuccgacgcuagggg	cggugagggagccugacuc	scaffold258420|size588:41..128:+
agi-miR-6874-5p-1	hsa-miR-6874-5p	uuggagcugagaccugc	guggucucgccagug	scaffold206060|size783:671..723:-
agi-miR-8962	eca-miR-8962	cagcaaggugcugccagca	cuggcaguguguucgcugug	scaffold5070|size272705:133485..133525:-
agi-miR-4592	pma-miR-4592	gcagcggcugcggagcca	guucugaggcuucacugccu	scaffold942|size487518:424754..424797:-
agi-miR8707	gra-miR8707	guaaaaacugugaacugaa	aaauucacgguuugcacuu	scaffold18092|size118932:95493..95565:+
agi-miR1450	ptc-miR1450	cucaaugguggcauuccug	ggcuugccuccaugaggu	scaffold2678|size352355:102305..102357:+
agi-miR-679	rno-miR-679	gaaggucuugcggaaggu	cugcugaagaacaguuccg	scaffold24691|size84996:41388..41446:-
agi-miR6459b	ptc-miR6459b	gcucaagcggguucaggag	ccucaaccccagcuc	scaffold25239|size81950:26999..27035:-
agi-miR-679	rno-miR-679	gaaggucuugcggaaggu	cucuccuucgcagaauucu	scaffold24691|size84996:41428..41488:-
agi-miR-4530	hsa-miR-4530	cccagcagccacuguguu	cacugugcuggaugggaa	scaffold309|size633887:417210..417273:-
agi-miR417	osa-miR417	caauguagcuuccagcagug	uggcugggagagugaagcagcugca	scaffold7510|size223178:185524..185599:-
agi-miR-4133-3p	cin-miR-4133-3p	aauguugucucuguaagca	cacucgcugacaacauucg	scaffold31128|size59019:17317..17363:-
agi-miR-3657	hsa-miR-3657	gguguccugcccuuuguacaca	uguuaggggcugcugggaccacg	scaffold47708|size23932:1102..1160:-
agi-miR-624-3p	hsa-miR-624-3p	cacaaggugcugccagca	agggcuggcaccucuugac	scaffold26643|size75817:63149..63189:+
agi-miR-4335	ssc-miR-4335	uugcccaggugacuacaga	ugcuagucaccaugggguucuu	scaffold5436|size263938:39064..39117:+
agi-miR-8205-3p	cel-miR-8205-3p	cuagccucaaggcuacaag	ccuagcuuugacaagcugagg	scaffold11869|size168372:86466..86514:+
agi-miR5723	bra-miR5723	uaugugcuuuucccuccu	gaggccaacacaugaa	scaffold27206|size73550:35348..35392:+
agi-miR-637	hsa-miR-637	gcugggggcugaucuggaugugaa	aacagccacagccuucuccagcuu	scaffold373097|size340:53..121:+
agi-miR2275c-5p	ata-miR2275c-5p	gguguuggauguucuggg	caagggcauucaaccuccuu	scaffold16050|size132764:95149..95195:-
agi-miR5167a-3p	bdi-miR5167a-3p	ccaaugacuuuuggggaa	ccuugaggaucauugguc	scaffold71275|size11244:433..480:-
agi-miR-490-3p	hsa-miR-490-3p	aaaccugggggauuuuuu	aaaguuccccggauuuuc	scaffold19865|size108545:91310..91379:+
agi-miR167c-5p	ath-miR167c-5p	aaagcugccaucugugccucu	agggcagauggaguuguaa	scaffold10306|size184842:145880..145962:-
agi-miR5157a-5p	osa-miR5157a-5p	aacuuuuugcuccuauuu	augggaugcaaauuuuuga	scaffold18476|size116628:55605..55673:+
agi-miR-4686	hsa-miR-4686	gaucugcucucauguuugaugcu	cauccacucagagagcauguucc	scaffold32233|size55583:25919..25980:+
agi-miR-6874-5p-2	hsa-miR-6874-5p	cuggagcuccaggguca	acccuucaagcuucuuua	scaffold980|size483094:257909..257957:+
agi-miR-4530	hsa-miR-4530	cccagcagcugcuguguu	cacagugcgugcuggucu	scaffold541|size569980:405207..405270:+
agi-miR-5596b-3p	cin-miR-5596b-3p	augguggguggugaguguu	cgcuccaucccuagga	scaffold6420|size242431:52928..52964:-
agi-miR-5390	aca-miR-5390	gaauuccgacgauccagg	aaguucgucccggauucuc	scaffold15143|size139212:97595..97633:+
agi-miR-2483-5p	bta-miR-2483-5p	agucaaccugguugauccug	gagagucaaccugguugaucc	scaffold40653|size34333:16162..16240:+
agi-miR-4061-3p	cin-miR-4061-3p	cacacucuccuuguggua	ccaugggguccaugugca	scaffold768|size516110:484311..484360:-

Supplementary Table 8. Continued

Provisional id	miRBase miRNA with the same seed	Consensus mature sequence	Consensus star sequence	Precursor coordinate
agi-miR535-3p	osa-miR535-3p	augcuuucacuuugagg	ucaaauuguggcguga	scaffold2813|size346490:271845..271923:+
agi-miR-281	ngi-miR-281	cucauggauuugguagcaua	ucuugccagauucaaggauu	scaffold4684|size282686:136320..136386:+
agi-miR5368	gma-miR5368	agacagucugccugagac	cuccccucaaacugucugu	scaffold4235|size296169:148685..148725:+
agi-miR156ab	gma-miR156ab	auuuaaguuccgaggcuu	gcuucgaaacuuaaaggu	scaffold190815|size874:258..301:-
agi-miR-9221	efu-miR-9221	gccaagauuguugaagcu	caucuuacaaucccggaca	scaffold2108|size383744:133807..133859:+
agi-miR-9276	efu-miR-9276	uuggagcaagcuguauug	guccagcuugcaccauca	scaffold217|size671764:468757..468796:+
agi-miR11024	seu-miR11024	gaauaauuagauugggccuuug	agggcaagucuggugcca	scaffold1268425|size142:25..82:+
agi-miR-7398j-3p	mdo-miR-7398j-3p	aaaccuuuggagacuuca	augucucucugaggucugu	scaffold1599|size420085:233950..233994:-
agi-miR-2584-3p	dsi-miR-2584-3p	uaagaggugcugccagca	caucaagcaccugaauuugcu	scaffold7628|size221165:136555..136629:-
agi-miR-2483-5p	bta-miR-2483-5p	agucaaccugguugauccug	gagaaucaaucugguugaucc	scaffold40653|size34333:16107..16185:+
agi-miR-10461b-3p	sfr-miR-10461b-3p	cgaaacuaugccugagaa	acauggcaugguguuuugac	scaffold20681|size103846:95860..95938:+
agi-miR-509-5p	hsa-miR-509-5p	uacugcagcuuccagcau	auaggaagcccuugugcu	scaffold6127|size248820:13972..14019:-
agi-miR-1266-5p	hsa-miR-1266-5p	gcucagggaaaguaucca	gauauuuuucugaaaag	scaffold4354|size292118:17532..17606:-
agi-miR-3045a	api-miR-3045a	aagaauaauaccugcauacau	guaugcaggauuuggcgaag	scaffold1748|size408955:49434..49487:-
agi-miR1023c-3p	ppt-miR1023c-3p	ggggaaucaagcuugua	uaagcuuuguucaaaau	scaffold21990|size97308:25323..25371:+
agi-miR-4533	hsa-miR-4533	gggaaggagaacaagagc	gcuuguucucagcauccccu	scaffold4321|size293229:239464..239534:+
agi-miR1064-3p	ppt-miR1064-3p	gggugauucgguagcauc	cgcuucuuggccgaauccuauu	scaffold1994|size390830:72421..72467:-
agi-miR-2538-3p	dps-miR-2538-3p	caaauuagauagugugca	uacacuguuuugggc	scaffold321894|size448:370..420:+
agi-miR7721-5p	bdi-miR7721-5p	ccgggauucgggagcauc	aguucaccaaguuucuaag	scaffold8714|size205402:188282..188359:-
agi-miR-688	mmu-miR-688	acgcaggcaggcaaaaaa	uuuuguuuugugauc	scaffold472931|size238:76..161:-
agi-miR1217-5p	ppt-miR1217-5p	ugguaucaucucuggagc	uucagaggacugaccaag	scaffold5432|size263970:212329..212378:-
agi-miR6222-3p	sbi-miR6222-3p	auagcugaaugaugcguc	cguacaugagguuaucu	scaffold13214|size155577:54943..55030:+
agi-miR8040-5p	stu-miR8040-5p	ccauaauuaauggggauu	uccucucuacuuaaugaggca	scaffold2320|size370156:196019..196097:+
agi-miR5147	osa-miR5147	acaacucugaaugucaaa	ugaaaaauuuagguugugu	scaffold36163|size44271:33541..33586:+
agi-miR-1932	mmu-miR-1932	uuugcggacacagauuggaugagua	ggagucgaguuuguggaaaggg	scaffold4276|size295004:242401..242484:+
agi-miR-1764-5p	gga-miR-1764-5p	acucaggcaugcaaaaaaa	uuuuucgugcugagaac	scaffold436741|size261:114..169:+
agi-miR-9307	efu-miR-9307	cucugcagcuuccagcag	gcuugauggccuugggaau	scaffold3234|size328444:95747..95805:+
agi-miR-2314	bta-miR-2314	agcccauggcaggagcug	gccugccaugggauca	scaffold48445|size23152:9385..9426:+
agi-miR1886h	stu-miR1886h	uuuuuacgaccagaaaugu	aauucucuucgucgaaacc	scaffold7967|size216490:112494..112555:+
agi-miR-661	hsa-miR-661	ugccugguuguaguuuu	gcuuaugaccuugcacc	scaffold3330|size325086:161052..161099:+
agi-miR-6552-5p	gga-miR-6552-5p	aucuguccacgaucggac	ccgaugggugggccugaauu	scaffold18077|size119031:68844..68895:-
agi-miR-3677-3p	hsa-miR-3677-3p	uucguggggguguaggguu	ccgugaaaaccccagcuuug	scaffold1196|size457578:194308..194352:-
agi-miR-9212	efu-miR-9212	ccagcauguccaccaguag	gcugggaaauaugcgauua	scaffold3822|size308102:33152..33195:-
agi-miR-6506-3p	hsa-miR-6506-3p	gcguaucaagauguauug	auguauuaugauuua	scaffold3102|size333698:247659..247714:+
agi-miR162-5p	zma-miR162-5p	gggcgcagagaauugcug	ggaaaaccuccauguccau	scaffold13001|size157597:18195..18245:-
agi-miR-96b-3p	sme-miR-96b-3p	aacauuuccucuguuucc	aaacaggggaaauguuaa	scaffold801657|size168:6..58:-
agi-miR-6558-5p	gga-miR-6558-5p	gagugaaaugccucgucau	gacgaggucguuguugcugau	scaffold2146408|size120:95..138:-
aginovel-mir-0001	-	cgccgacacuuuggccga	gcccagaauugucggcaga	scaffold26709|size75566:73734..73795:-
aginovel-mir-0002	-	aaaagagugcuucggcuu	guaguaucuuggga	scaffold132|size740999:628628..628676:+
aginovel-mir-0003	-	guagcacggugggugcca	ucgcgcuacacgugucgcag	scaffold1929|size394924:162534..162605:-
aginovel-mir-0004	-	acggaggcaugcaaaaaa	uugugcauuguuucugugg	scaffold25366|size81326:54676..54730:-
aginovel-mir-0005	-	ugugcacucuaguguauu	uacacaggggcgguugugcgac	scaffold24696|size84366:73878..73922:-
aginovel-mir-0006	-	gcgugugagcccugauaa	acuagggcuuacuaaagcug	scaffold13137|size156250:113685..113763:+

Supplementary Table 8. Continued

Provisional id	miRBase miRNA with the same seed	Consensus mature sequence	Consensus star sequence	Precursor coordinate
aginovel-mir-0007	-	uuccagauuugggagauu	uuuucuggguugguggaaau	scaffold12567|size161594:85612..85679:-
aginovel-mir-0008	-	gcgugugagcccugauaa	gucacuacacagguca	scaffold13137|size156250:113647..113703:+
aginovel-mir-0009	-	aagccuuggcacugcuac	ugucauugccacgcuuug	scaffold6842|size234363:94785..94822:+
aginovel-mir-0010	-	acgcaaaagaguaguguu	cauccuguuuucuuucuguuu	scaffold2236|size375360:285337..285423:+
aginovel-mir-0011	-	uugcguuucacagcaagg	cagcugucauacgcuugu	scaffold14345|size145649:144986..145053:+
aginovel-mir-0012	-	cccgauucgggcgcauc	uuuguccuagucccaaa	scaffold2565|size357887:91449..91491:+
aginovel-mir-0013	-	guaguggcucugagggcu	cagggucggggcggcucugacuu	scaffold16534|size129381:32156..32242:-
aginovel-mir-0014	-	gucgaaacaggacauguc	uauguccucuucuguccc	scaffold15654|size135393:36137..36211:+
aginovel-mir-0015	-	gcgcgcaaacugugaacugaa	cacuucacgguuuagcccc	scaffold536|size572684:30075..30158:+
aginovel-mir-0016	-	cuagcuguccaaggucaa	gaccucuuagcaaaaauggau	scaffold32564|size54527:52703..52762:+
aginovel-mir-0017	-	ggacaccaacaagaucuga	uugucuugauaguccuu	scaffold4624|size284481:94074..94122:+
aginovel-mir-0018	-	auauaugggcuguaguguu	aacuacauguauauauau	scaffold7505|size223239:182727..182778:-
aginovel-mir-0019	-	caguaggcaugcaaaaaaa	uguguguugucuacucuc	scaffold18447|size116799:77737..77790:+
aginovel-mir-0020	-	augaauaauaccugcauacau	guaugcagguauuggagaaga	scaffold224898|size692:22..76:-
aginovel-mir-0021	-	cuaauuaauggggauugu	aauucucacuaauuagug	scaffold2224|size375983:296934..296989:+
aginovel-mir-0022	-	guagcagaucugaacaaa	uuuaaaggucauugccuuugcgg	scaffold18490|size116526:88084..88150:-
aginovel-mir-0023	-	guagguuaaaaagcucg	ugcuuauuagaugcag	scaffold525|size576073:373641..373704:-

Supplementary Table 9. Main differential expressed genes during nutrient starvation in *A. gibbosum* as shown on Fig. 3.

Abbreviation	Details
AAT	Amino acid transporters
ABC	ABC transporter
AMT	Ammonium transporter
ANK3	Ankyrin repeat proteins
APD	Acyl-CoA dehydrogenase
BCAT	Branch chain aminotransferase
BAND3	Band 3 anion transport protein
Cyt-b	Cytochrome b
Cyt-C	Cytochrome C
CYP 455	Cytochrome P450
COX	Cytochrome c oxidase
CYBR5	Cytochrome b5 reductase 3
CYTC	Cytochrome c-550
DUR	Urea-proton symporter
FRDB	Fumarate reductase
GAPCP	Glyceraldehyde-3-phosphate dehydrogenase (chloroplastic)
GK	Glucokinase
G3PD	Glyceraldehyde-3-phosphate dehydrogenase
HMA5	Copper-transporting ATPase
GMPS	GMP synthase
GSII	Glutamine synthetase
LRR	LRR receptor-like serine/threonine-protein kinase
MPT	Mitochondrial phosphate carrier protein
NIA	Nitrate reductase
NIR	Nitrite reductase
NRT	Nitrate transporter
NRAMP	Metal transporter
PCP	Peridinin-chlorophyll a-binding protein
PDK	Pyruvate dehydrogenase
PER	Adenine/guanine permease
PK	Pyruvate kinase
PMPCB	Probable mitochondrial-processing peptidase
PRX2D	Peroxiredoxin-2D
PRX2E	Peroxiredoxin-2E
PSAA	Photosystem I P700 chlorophyll a
PSAT	Phosphoserine aminotransferase
RBC	Ribulose bisphosphate carboxylase
STP	Serine/threonine-protein phosphatase
TPT	Phosphate translocator
ZIP	Zinc transporter

Supplementary Table 10. Annotation of PKS and NRPS genes under nitrogen and phosphate starvation, as shown in Fig. 3b.

ID	Annotation	sampleA	sampleB	logFC	logCPM	PValue	FDR	P1	P2	P3	NC1	NC2	NC3
TRINITY_DN103845_c0_g1	Enoylreductase	Control	Phosphate	-13.99652335	10.45221477	0	0	19.316	19.033	22.314	0	0	0
TRINITY_DN112574_c0_g1	Ketoreductase	Control	Phosphate	-11.90633733	9.904171143	0	0	18.577	16.382	18.304	0	0	0.008
TRINITY_DN134547_c0_g1	AMP-binding	Control	Phosphate	-11.80367469	10.57277446	0	0	10.12	9.301	10.672	0	0	0.008
TRINITY_DN129964_c0_g1	Acyltransferase	Control	Phosphate	-10.76100638	12.62138344	0	0	32.221	30.577	35.148	0.018	0.009	0.042
TRINITY_DN162352_c0_g1	Enoylreductase	Control	Phosphate	-10.51761681	9.724232699	0	0	13.089	11.918	13.243	0	0	0.034
TRINITY_DN134010_c0_g7	Ketoreductase	Control	Phosphate	-10.46738693	11.02092838	0	0	41.382	33.001	39.508	0.026	0	0.068
TRINITY_DN133872_c0_g1	AMP-binding	Control	Phosphate	-10.2904743	12.52039777	0	0	44.998	38.553	49.034	0.132	0	0.068
TRINITY_DN130988_c0_g3	AMP-binding	Control	Phosphate	-10.25486782	10.61068097	0	0	15.596	15.419	17.171	0.009	0	0.042
TRINITY_DN134436_c0_g4	PP-binding	Control	Phosphate	-10.19991104	10.73938748	0	0	6.874	6.004	6.207	0.009	0	0.008
TRINITY_DN105906_c0_g4	Enoylreductase	Control	Phosphate	-10.06535053	10.19039246	0	0	12.154	10.763	11.771	0.035	0	0.017
TRINITY_DN74309_c0_g1	Ketoreductase	Control	Phosphate	-9.974886787	11.40403768	0	0	43.508	44.919	45.2	0.115	0	0.025
TRINITY_DN81408_c0_g1	Dehydratase	Control	Phosphate	-9.8780143	11.20824385	0	0	47.979	43.265	45.925	0.044	0.009	0.136
TRINITY_DN83583_c0_g2	Thioesterase	Control	Phosphate	-9.876204301	9.991334526	0	0	9.138	8.553	9.912	0.009	0.009	0.017
TRINITY_DN124802_c0_g1	Enoylreductase	Control	Phosphate	-9.808896398	10.14269349	0	0	18.392	16.676	19.31	0	0	0.085
TRINITY_DN84020_c0_g1	Enoylreductase	Control	Phosphate	-9.776597712	10.99371914	0	0	16.486	16.994	18.129	0.035	0.009	0.025
TRINITY_DN134010_c0_g6	Ketoreductase	Control	Phosphate	-9.76403799	11.60590949	0	0	34.254	30.622	32.424	0.026	0.017	0.093
TRINITY_DN135178_c0_g1	Acyltransferase	Control	Phosphate	-9.645295791	12.16341945	0	0	47.771	44.863	46.427	0.079	0.026	0.102
TRINITY_DN115752_c0_g1	Acyltransferase	Control	Phosphate	-9.569371968	10.40363096	0	0	11.172	9.845	11.993	0.009	0	0.051
TRINITY_DN123321_c0_g1	Ketosynthase	Control	Phosphate	-9.509838032	10.33367262	0	0	4.147	3.614	4.196	0.018	0	0
TRINITY_DN77651_c0_g1	Enoylreductase	Control	Phosphate	-9.507274009	10.82323254	0	0	22.181	18.33	20.829	0.026	0.026	0.051
TRINITY_DN132744_c0_g1	AMP-binding	Control	Phosphate	-9.478681128	12.80816534	0	0	69.039	63.918	66.684	0.132	0.009	0.178
TRINITY_DN123354_c0_g1	Enoylreductase	Control	Phosphate	-9.423488633	11.79771148	0	0	51.144	45.135	57.321	0.079	0.026	0.288
TRINITY_DN107054_c0_g3	Ketoreductase	Control	Phosphate	-9.135878711	11.28732272	0	0	26.248	24.878	27.913	0.026	0.009	0.144
TRINITY_DN101594_c0_g1	Ketosynthase	Control	Phosphate	-9.003987627	9.939227661	0	0	10.444	9.358	11.595	0.053	0	0.017
TRINITY_DN100180_c0_g1	Ketoreductase	Control	Phosphate	-8.350928709	10.28317435	0	0	17.618	16.971	21.18	0.079	0	0.136
TRINITY_DN132537_c1_g4	Enoylreductase	Control	Phosphate	-8.33231772	10.52182193	0	0	13.979	12.099	13.699	0.009	0.009	0.161
TRINITY_DN116546_c0_g3	AMP-binding	Control	Phosphate	-6.779194794	10.80884606	0	0	12.824	12.009	13.781	0.38	0.218	0.297
TRINITY_DN134873_c1_g4	Ketosynthase	Control	Phosphate	-5.771116166	10.65130937	0	0	7.186	7.024	8.077	1.483	1.204	0.933
TRINITY_DN130988_c0_g1	AMP-binding	Control	Phosphate	-9.369559691	9.872050165	6.4251955 1149037e-310	0.00E+00	9.612	7.84	7.574	0.009	0	0.042
TRINITY_DN93390_c0_g1	Enoylreductase	Control	Phosphate	-13.18978671	9.595993149	2.19E-304	3.87E-303	8.445	6.899	8.392	0	0	0
TRINITY_DN101387_c0_g1	Enoylreductase	Control	Phosphate	-12.95997686	9.343426756	2.53E-302	4.32E-301	24.111	24.437	24.266	0	0	0
TRINITY_DN127874_c0_g2	AMP-binding	Control	Phosphate	-9.896597671	9.449736302	8.83E-292	1.46E-290	7.301	6.038	7.06	0	0	0.025
TRINITY_DN82050_c0_g1	Ketosynthase	Control	Phosphate	-9.332375153	9.149647242	4.53E-274	7.26E-273	9.519	8.814	9.783	0.009	0	0.042
TRINITY_DN104085_c0_g2	Enoylreductase	Control	Phosphate	-8.859777852	9.114482238	1.26E-268	1.96E-267	11.726	11.612	11.595	0.053	0.017	0
TRINITY_DN88509_c0_g1	Thioesterase	Control	Phosphate	-10.39546658	9.063271238	3.82E-267	5.77E-266	21.604	21.027	23.775	0	0.035	0
TRINITY_DN127136_c0_g1	Ketosynthase	Control	Phosphate	-8.53732244	9.29626224	4.91E-252	7.21E-251	3.085	3.172	2.782	0.018	0	0.017
TRINITY_DN114507_c2_g3	Thioesterase	Control	Phosphate	-8.184179478	9.414136449	2.72E-251	3.89E-250	4.69	4.09	3.729	0.018	0.017	0.008
TRINITY_DN119609_c0_g3	PP-binding	Control	Phosphate	-9.30356422	9.378128258	2.47E-249	3.44E-248	10.79	9.686	8.638	0.026	0	0.034
TRINITY_DN137156_c0_g1	Thioesterase	Control	Phosphate	-10.87398451	9.582795814	4.63E-248	6.29E-247	3.35	2.878	2.56	0	0	0
TRINITY_DN70633_c0_g2	Ketosynthase	Control	Phosphate	-8.399347066	8.998211712	6.14E-242	8.12E-241	4.598	3.841	4.161	0.009	0	0.034
TRINITY_DN65223_c0_g1	AMP-binding	Control	Phosphate	-12.38684465	8.717980718	3.49E-228	4.51E-227	10.825	10.536	11.057	0	0	0

TRINITY_DN136849_c0_g5	Ketosynthase	Control	Phosphate	-10.68486879	8.581803633	7.42E-195	9.35E-194	4.413	3.625	4.383	0	0	0.008
TRINITY_DN73152_c0_g1	Ketosynthase	Control	Phosphate	-5.959631922	9.131104508	2.40E-193	2.95E-192	16.139	11.805	14.798	0.283	0.271	0.204
TRINITY_DN134045_c1_g1	Dehydratase	Control	Phosphate	-10.55477438	8.433997891	1.47E-190	1.77E-189	4.783	4.69	5.388	0	0	0.008
TRINITY_DN98117_c0_g2	Enoylreductase	Control	Phosphate	-12.28855182	8.610350911	2.15E-175	2.53E-174	12.442	12.96	16.119	0	0	0
TRINITY_DN32533_c0_g1	Ketosynthase	Control	Phosphate	-8.462067455	8.191960692	5.43E-161	6.25E-160	5.407	4.792	4.711	0.035	0	0.008
TRINITY_DN135661_c1_g3	Thioesterase	Control	Phosphate	-2.723842996	11.44367472	2.31E-157	2.60E-156	18.057	18.058	19.614	3.673	4.704	5.268
TRINITY_DN131164_c0_g1	PP-binding	Control	Phosphate	-11.91054239	8.20055196	6.39E-149	7.04E-148	1.802	1.45	1.999	0	0	0
TRINITY_DN117223_c0_g2	Enoylreductase	Control	Phosphate	-11.98824598	8.284164488	8.76E-149	9.46E-148	17.872	14.082	13.828	0	0	0
TRINITY_DN55783_c0_g1	Enoylreductase	Control	Phosphate	-11.71472892	7.973115982	3.37E-144	3.56E-143	3.466	3.569	3.483	0	0	0
TRINITY_DN84105_c1_g1	Enoylreductase	Control	Phosphate	-8.426892174	7.831565558	5.80E-135	6.02E-134	2.669	2.447	2.525	0.018	0	0
TRINITY_DN78078_c0_g1	Thioesterase	Control	Phosphate	-10.00377641	7.830853761	1.39E-131	1.41E-130	3.258	2.787	3.331	0	0	0.008
TRINITY_DN15499_c0_g2	AMP-binding	Control	Phosphate	-8.310398203	8.043706556	4.19E-130	4.18E-129	2.761	2.288	3.214	0	0	0.034
TRINITY_DN49996_c0_g1	Ketosynthase	Control	Phosphate	-10.17188269	8.016929448	2.01E-128	1.97E-127	2.669	2.119	2.069	0	0	0.008
TRINITY_DN77380_c0_g1	AMP-binding	Control	Phosphate	-8.384865253	7.790649253	1.64E-127	1.57E-126	8.018	7.171	8.743	0.035	0.017	0.025
TRINITY_DN123261_c0_g6	Ketoreductase	Control	Phosphate	-9.01209216	8.090650767	1.22E-126	1.15E-125	3.35	3.365	4.547	0	0	0.025
TRINITY_DN11291_c0_g1	Enoylreductase	Control	Phosphate	-11.598867	7.839642289	1.45E-121	1.35E-120	3.004	3.319	2.747	0	0	0
TRINITY_DN103908_c0_g1	Ketoreductase	Control	Phosphate	-9.765532682	7.559656212	2.29E-112	2.09E-111	3.212	3.217	3.413	0.009	0	0
TRINITY_DN102380_c0_g2	Ketoreductase	Control	Phosphate	-11.24252918	7.45374159	2.47E-106	2.22E-105	3.096	3.127	3.238	0	0	0
TRINITY_DN116334_c0_g1	Ketosynthase	Control	Phosphate	-11.45091859	7.689306825	1.83E-104	1.61E-103	4.251	3.217	3.191	0	0	0
TRINITY_DN87066_c0_g1	Ketosynthase	Control	Phosphate	-11.35458812	7.571509258	2.89E-104	2.51E-103	2.126	2.266	2.081	0	0	0
TRINITY_DN89507_c0_g1	AMP-binding	Control	Phosphate	-8.957021084	7.473914102	2.93E-103	2.50E-102	6.989	7.749	7.095	0.035	0	0
TRINITY_DN89999_c0_g1	Thioesterase	Control	Phosphate	-11.02479335	7.219892044	9.94E-88	8.34E-87	2.542	2.277	2.42	0	0	0
TRINITY_DN136021_c1_g4	Ketoreductase	Control	Phosphate	-11.18801279	7.405839199	3.40E-86	2.81E-85	3.189	2.39	2.525	0	0	0
TRINITY_DN104831_c0_g1	Thioesterase	Control	Phosphate	-11.06758669	7.270272474	3.85E-85	3.13E-84	2.449	2.175	2.817	0	0	0
TRINITY_DN136954_c0_g3	AMP-binding	Control	Phosphate	-8.707744256	7.219971596	1.08E-72	8.70E-72	5.568	4.033	4.114	0.026	0	0
TRINITY_DN44060_c0_g1	AMP-binding	Control	Phosphate	-10.62843324	6.78175689	8.34E-70	6.59E-69	10.501	11.646	10.707	0	0	0
TRINITY_DN68351_c0_g1	Thioesterase	Control	Phosphate	-7.138621413	7.021867717	3.81E-67	2.96E-66	2.599	1.79	2.536	0.035	0.017	0
TRINITY_DN89999_c0_g3	Thioesterase	Control	Phosphate	-8.889529647	6.617217999	9.38E-66	7.19E-65	2.334	2.322	2.688	0	0	0.017
TRINITY_DN131734_c0_g3	Ketosynthase	Control	Phosphate	-1.653481563	10.34999777	1.00E-63	7.56E-63	9.057	8.678	9.585	3.735	3.919	4.623
TRINITY_DN123216_c0_g1	AMP-binding	Control	Phosphate	-8.486170493	6.952918907	1.68E-63	1.25E-62	2.103	2.866	2.934	0.018	0	0
TRINITY_DN137808_c0_g4	AMP-binding	Control	Phosphate	-8.784919627	14.54738955	2.20E-59	1.61E-58	246.975	232.335	241.909	0.795	1.143	1.773
TRINITY_DN102073_c0_g1	AMP-binding	Control	Phosphate	-10.2070173	6.341208019	2.08E-57	1.51E-56	0.947	0.872	0.853	0	0	0
TRINITY_DN100553_c0_g1	Thioesterase	Control	Phosphate	-6.755221201	6.374489641	5.84E-57	4.17E-56	2.923	2.617	2.782	0	0	0.102
TRINITY_DN63251_c0_g1	Ketosynthase	Control	Phosphate	-10.20157404	6.327662208	1.79E-56	1.26E-55	1.19	1.325	1.286	0	0	0
TRINITY_DN102799_c0_g2	AMP-binding	Control	Phosphate	-10.21834069	6.35193997	3.83E-54	2.66E-53	2.888	2.628	2.244	0	0	0
TRINITY_DN131600_c0_g1	Ketoreductase	Control	Phosphate	-1.202333269	10.93464062	6.55E-53	4.50E-52	15.03	14.048	16.329	7.037	7.803	6.82
TRINITY_DN102799_c0_g1	AMP-binding	Control	Phosphate	-10.27236129	6.4085886	1.26E-52	8.54E-52	1.248	1.11	0.912	0	0	0
TRINITY_DN130988_c0_g2	Thioesterase	Control	Phosphate	-10.5028579	6.670234615	6.15E-52	4.07E-51	3.535	2.844	4.839	0	0	0
TRINITY_DN89056_c0_g1	Enoylreductase	Control	Phosphate	-10.2742821	6.405497063	2.20E-48	1.44E-47	1.236	1.325	1.847	0	0	0
TRINITY_DN136279_c0_g1	AMP-binding	Control	Phosphate	-9.974017004	6.077291573	1.46E-44	9.28E-44	0.866	1.122	0.83	0	0	0
TRINITY_DN134862_c1_g3	AMP-binding	Control	Phosphate	-8.078444709	5.772483542	1.76E-44	1.11E-43	2.403	2.22	2.384	0	0.017	0
TRINITY_DN58117_c0_g1	Thioesterase	Control	Phosphate	-9.695489907	5.823234596	5.01E-41	3.12E-40	0.809	0.668	1.005	0	0	0
TRINITY_DN106618_c0_g1	Ketosynthase	Control	Phosphate	-7.372618824	5.795923313	7.55E-41	4.65E-40	0.589	0.623	0.479	0.009	0	0

TRINITY_DN125765_c1_g2	PP-binding	Control	Phosphate	-1.322354075	11.00717255	5.08E-39	3.05E-38	22.285	23.383	27.305	12.282	13.031	14.539
TRINITY_DN116730_c0_g2	PP-binding	Control	Phosphate	-9.585612223	5.717759327	1.65E-37	9.81E-37	1.756	1.144	1.181	0	0	0
TRINITY_DN128274_c5_g3	Thioesterase	Control	Phosphate	-9.416729387	5.540444913	6.59E-37	3.87E-36	0.855	0.657	0.795	0	0	0
TRINITY_DN75812_c0_g3	AMP-binding	Control	Phosphate	-9.291220986	5.423588681	6.76E-33	3.93E-32	3.05	2.085	2.735	0	0	0
TRINITY_DN101514_c0_g3	PP-binding	Control	Phosphate	-9.50855835	5.625245328	1.03E-31	5.92E-31	0.751	0.612	0.432	0	0	0
TRINITY_DN137134_c2_g5	AMP-binding	Control	Phosphate	-8.666901411	4.825530722	4.04E-23	2.27E-22	1.848	1.178	1.718	0	0	0
TRINITY_DN132069_c0_g3	Ketoreductase	Control	Phosphate	1.179976268	11.08140255	4.38E-19	2.41E-18	50.821	41.883	40.186	129.802	104.96	84.315
TRINITY_DN44563_c0_g1	Ketosynthase	Control	Phosphate	-8.305794154	4.450124759	4.71E-18	2.57E-17	0.855	1.303	0.807	0	0	0
TRINITY_DN84045_c0_g1	AMP-binding	Control	Phosphate	-8.183836598	4.342887899	3.75E-16	2.00E-15	0.543	0.702	0.351	0	0	0
TRINITY_DN115500_c0_g2	AMP-binding	Control	Phosphate	-3.877611183	5.337775107	1.19E-14	6.28E-14	0.89	0.748	0.643	0.053	0.07	0.263
TRINITY_DN50568_c0_g1	AMP-binding	Control	Phosphate	-7.638257715	3.895047815	4.05E-12	1.95E-11	0.531	0.464	0.245	0	0	0
TRINITY_DN90979_c0_g1	Thioesterase	Control	Phosphate	-6.985269202	3.402528732	3.25E-07	1.41E-06	2.079	1.156	0.327	0	0	0
TRINITY_DN8230_c0_g2	Condensation	Control	Phosphate	-6.521929563	3.058595006	9.56E-07	4.05E-06	0.716	0.691	0.374	0	0	0
TRINITY_DN135421_c6_g4	PP-binding	Control	Phosphate	-6.630438365	3.102292007	9.74E-07	4.09E-06	0.254	1.915	1.145	0	0	0
TRINITY_DN109686_c0_g2	AMP-binding	Control	Phosphate	-2.233345345	6.449858155	1.05E-06	4.39E-06	1.098	0.906	0.947	0.132	0.454	0.195
TRINITY_DN135180_c8_g5	Ketosynthase	Control	Phosphate	-6.392357257	2.969942225	4.95E-06	1.95E-05	0.774	0.827	0.362	0	0	0
TRINITY_DN94025_c0_g2	Thioesterase	Control	Phosphate	-6.120691077	2.824988779	3.13E-05	0.000115793	0.393	0.147	0.479	0	0	0
TRINITY_DN137396_c0_g1	AMP-binding	Control	Phosphate	1.150231245	5.861280262	0.000461496	0.001453162	0.092	0.102	0.152	0.318	0.314	0.271
TRINITY_DN106210_c0_g2	Enoylreductase	Control	Phosphate	3.068119961	4.844178715	0.001040087	0.003108508	0	0	0.105	0.583	0	0.433
TRINITY_DN191271_c0_g1	Thioesterase	Control	Phosphate	-5.423483414	2.422808869	0.001272995	0.00370008	0.058	0.147	0.117	0	0	0
TRINITY_DN62730_c0_g2	Thioesterase	Control	Phosphate	-1.08534518	7.352054445	0.001338384	0.003847854	1.282	0.793	1.414	0.627	0.367	0.865
TRINITY_DN112164_c0_g1	Ketoreductase	Control	Phosphate	1.135100635	6.258511928	0.001358325	0.003884074	0.347	0.227	0.187	0.556	0.506	0.84
TRINITY_DN144450_c0_g1	Ketosynthase	Control	Phosphate	2.373358744	3.196626759	0.012676241	0.030205998	0	0	0.07	0.238	0.122	0.102
TRINITY_DN59021_c0_g1	Dehydratase	Control	Phosphate	1.294391263	4.823393426	0.021351169	0.048062844	0.069	0	0.035	0.097	0.096	0.11

ID	Annotation	sampleA	sampleB	logFC	logCPM	PValue	FDR	N1	N2	N3	NC1	NC2	NC3
TRINITY_DN112164_c0_g1	Ketoreductase	Control	Nitrate	1.770227457	6.174593481	3.24E-06	4.24E-05	0.186	0.205	0.143	0.623	0.565	0.953
TRINITY_DN103845_c0_g1	Enoylreductase	Control	Nitrate	-6.896073507	3.524443534	1.15E-05	0.000114449	0	0.026	0.01	0	0	0
TRINITY_DN136849_c0_g3	Ketosynthase	Control	Nitrate	3.122103587	4.557844188	0.00015628	0.001006689	0.072	0.041	0.194	0.168	0.682	0.857
TRINITY_DN93390_c0_g1	Enoylreductase	Control	Nitrate	-5.98262817	2.875986283	0.00044566	0.002600557	0	0.082	0.01	0	0	0
TRINITY_DN132934_c2_g4	PP-binding	Control	Nitrate	2.125876168	4.350828013	0.000763411	0.004071525	0.062	0.308	0.112	0.998	1.179	0.905
TRINITY_DN203644_c0_g1	Dehydratase	Control	Nitrate	2.880567147	3.448979342	0.002062218	0.009214955	0.021	0.01	0.132	0.148	0.127	0.106
TRINITY_DN112574_c0_g1	Ketoreductase	Control	Nitrate	-4.08135833	2.830119463	0.00210949	0.009342026	0.072	0.123	0.01	0	0	0.01
TRINITY_DN84020_c0_g1	Enoylreductase	Control	Nitrate	-2.002174381	3.901566367	0.003848331	0.014912284	0.062	0.113	0.051	0.04	0.01	0.029
TRINITY_DN59021_c0_g1	Enoylreductase	Control	Nitrate	1.438625474	4.815299681	0.004747157	0.017837801	0.021	0.051	0.061	0.109	0.107	0.125
TRINITY_DN82032_c0_g1	Thioesterase	Control	Nitrate	5.04804279	2.567793821	0.006272702	0.022709929	0	0	0.041	0.04	0.049	0.029
TRINITY_DN129964_c0_g1	Acyltransferase	Control	Nitrate	-2.424625523	4.798395564	0.006872082	0.024641751	0.031	0.087	0.081	0.02	0.01	0.048
TRINITY_DN137808_c0_g4	AMP-binding	Control	Nitrate	-1.66429315	7.92392674	0.009084966	0.030242572	1.249	1.36	1.344	0.89	1.276	1.012
TRINITY_DN134547_c0_g1	AMP-binding	Control	Nitrate	-3.264439834	2.8758553	0.011054783	0.035605017	0	0.051	0	0	0	0.01
TRINITY_DN137213_c4_g2	Ketosynthase	Control	Nitrate	1.405960013	5.586706721	0.011890795	0.038050543	0.279	0.246	0.662	0.563	1.111	0.982
TRINITY_DN77651_c0_g1	Enoylreductase	Control	Nitrate	-2.225531841	4.16770995	0.013768302	0.043222012	0	0.046	0.061	0.03	0.029	0.058
TRINITY_DN135178_c0_g1	Acyltransferase	Control	Nitrate	-1.839036521	4.955186947	0.016638414	0.049959461	0.052	0.159	0.122	0.089	0.029	0.116
